# Variable Selection with FDR Control for Noisy Data – An Application to Screening Metabolites that Are Associated with Breast Cancer and Colorectal Cancer

**DOI:** 10.6339/25-jds1166

**Published:** 2025-06-11

**Authors:** Runqiu Wang, Ran Dai, Ying Huang, Marian L. Neuhouser, Johanna W. Lampe, Daniel Raftery, Fred K. Tabung, Cheng Zheng

**Affiliations:** 1 Department of Biostatistics, University of Nebraska Medical Center, Omaha, Nebraska, U.S.A.; 2 Division of Public Health Sciences, Fred Hutchinson Cancer Center, Seattle, Washington, U.S.A.; 3 Department of Anesthesiology and Pain Medicine, University of Washington, Seattle, Washington, U.S.A.; 4 Department of Internal Medicine, College of Medicine and Comprehensive Cancer Center, The Ohio State University, Columbus, Ohio, U.S.A.

**Keywords:** cancer, FDR control, measurement error, metabolomics data, missing data, variable selection

## Abstract

The rapidly expanding field of metabolomics presents an invaluable resource for understanding the associations between metabolites and various diseases. However, the high dimensionality, presence of missing values, and measurement errors associated with metabolomics data can present challenges in developing reliable and reproducible approaches for disease association studies. Therefore, there is a compelling need for robust statistical analyses that can navigate these complexities to achieve reliable and reproducible disease association studies. In this paper, we construct algorithms to perform variable selection for noisy data and control the False Discovery Rate when selecting mutual metabolomic predictors for multiple disease outcomes. We illustrate the versatility and performance of this procedure in a variety of scenarios, dealing with missing data and measurement errors. As a specific application of this novel methodology, we target two of the most prevalent cancers among US women: breast cancer and colorectal cancer. By applying our method to the Women’s Health Initiative data, we successfully identify metabolites that are associated with either or both of these cancers, demonstrating the practical utility and potential of our method in identifying consistent risk factors and understanding shared mechanisms between diseases.

## 1 Introduction

Breast cancer (BC) and colorectal cancer (CRC) have a high incidence rate, ranking as the highest and third highest among women in the US, respectively (ACS, 2020). Both cancers share several diet and lifestyle risk factors (Kampman et al., 2018). According to the World Cancer Research Fund (WCRF)/American Institute for Cancer Research (AICR) Expert Panel, there is “convincing” evidence that adult weight gain and excess body fat increase the risk for post-menopausal BC and CRC, and that physical activity reduces the risk for both cancers. Furthermore, alcoholic drinks have been found to increase the risk of post-menopausal BC and CRC. Higher intakes of red meat, animal fats, and refined carbohydrates have been associated with increased risks of both BC and CRC, whereas fruits, vegetables, whole grains, and dietary fiber tend to be linked with reduced risk (Yusof et al., 2012; Xiao et al., 2019; Putri et al., 2013). However, the WCRF/AICR Expert Panel’s classification for the level of evidence supporting the associations for these dietary components remains “suggestive” or “probable” rather than “convincing”, except for increased intakes of processed meat and the risk of CRC (Kampman et al., 2018). Given this context, there is a crucial need to identify “convincing” evidence for risk factors associated with BC and CRC. Additionally, it is essential to study the common risk factors for these two prevalent cancers to better understand their shared underlying mechanisms and develop effective prevention strategies.

Metabolomics, the extensive analysis of small molecules in organisms (Nannini et al., 2020), reflects both internal cellular processes and external exposures, making it a sensitive tool for tracing pathways associated with chronic diseases like cancer. Despite its use in early cancer detection (Cheung et al., 2019; Yang et al., 2020; His et al., 2019; Zhu et al., 2014), few studies have systematically explored metabolomics in relation to BC and CRC. Identifying metabolomic components could uncover new BC pathways. For example, studies using the European Prospective Investigation into Cancer (EPIC) and the Prospective Lung, Colorectal, and Ovarian Cancer (PLCO) cohorts found certain plasma components and pre-diagnostic diet-related metabolites significantly associated with BC risk (His et al., 2019; Playdon et al., 2017). Further uncovering BC and CRC-related features could illuminate disease-related biological pathways, enhancing prevention and treatment strategies.

Developing screening methods for metabolomics data presents several challenges due to their inherent characteristics. Metabolomics data are high-dimensional, often containing missing values and measurement errors. The high dimensionality of metabolomics data is a double-edged sword: while it encompasses all potential components associated with the disease, the majority of these components are unrelated, introducing a significant amount of noise. Missingness and measurement errors are inevitable when dealing with large-scale data collection. These factors pose considerable challenges in screening procedures for the metabolomics data that offer reproducibility guarantees. To address these issues, robust and innovative approaches must be designed, which can account for the complexities and limitations associated with high-dimensional, noisy data while still providing accurate and reliable results.

Measurement errors and missing data frequently arise in complex data analysis tasks, posing challenges that must be carefully addressed when designing variable selection procedures. Naive approaches often lead to problematic results. For example, using complete case analysis and removing all samples with any missing data leads to spurious results in variable selection. Measurement errors lead to inflated estimation errors for coefficients and inconsistency in the Lasso variable selection procedure (Sorensen et al., 2015). There has been a surge in the literature on variable selection in the presence of missing data and measurement errors. For missing data mechanisms like missing at random (MAR) and missing completely at random (MCAR), researchers have developed imputation-based methods and other techniques (Little and Rubin, 2002; Tsiatis, 2006; Rässler et al., 2013) tailored for variable selection purposes (Wolfson, 2011; Johnson, 2008; Garcia et al., 2010). In the context of variable selection with measurement errors, several methods have been proposed, including CocoLasso (Datta and Zou, 2017), corrected Lasso (Loh and Wainwright, 2012; Sorensen et al., 2015), generalized matrix uncertainty selector (Rosenbaum and Tsybakov, 2013), generalized matrix uncertainty Lasso (Sorensen et al., 2015), and generalized Dantzig selector (Antoniadis et al., 2010). These advancements demonstrate the ongoing efforts to tackle the challenges posed by missing data and measurement errors in variable selection.

One critical challenge in variable selection is ensuring replicability guarantees. Over the past few decades, a novel measure for Type I error, the False Discovery Rate (FDR), or the expectation of the false discovery proportion (FDP), has been proposed to address this issue. The renowned Benjamini-Hochberg (BH) procedure (Benjamini and Hochberg, 1995) has sparked a new era in multiple hypothesis testing, leading to the rapid development of methods that control FDR. Among these techniques, the Knockoff-based methods (Barber and Candès, 2015, 2019; Candès et al., 2018) offer several advantages, making it particularly attractive for various applications. Some of the key benefits of the Knockoff methods include: mild assumptions about data structure, allowing for more flexibility in handling diverse datasets; compatibility with a wide array of models and variable selection procedures for both low and high dimensional data, powerful method with a finite sample FDR control guarantee, providing reliable results even with limited sample sizes.

These advantages make the Knockoff methods especially suitable for applications in metabolomic data, which typically exhibit complex correlation structures, high-dimensional features, and unknown signal strength. Furthermore, the Knockoff methods excel at handling arbitrary correlation structures and do not require prior knowledge of signal amplitudes or noise levels, making them powerful and versatile tools in the realm of variable selection.

### 1.1 Prior Work

#### Knockoff-Based Methods

Advancements in multiple testing problems within a single experiment have resulted in the development of powerful knockoff-based methods that provide exact FDR control for selecting features with conditional associations with the response (Barber and Candès, 2015; Candès et al., 2018). The knockoff filter by Barber and Candès (2015) offers exact FDR control for linear models without needing detailed model information and has been developed for high-dimensional cases (Barber and Candès, 2019). The Model-X knockoff by Candès et al. (2018) extends this to nonlinear models with unknown response distributions but requires knowledge of the predictor X’s distribution. Barber et al. (2020) demonstrated that the Model-X knockoff method is robust to errors in estimating the distribution of X, while Huang and Janson (2020) relaxed its assumptions, allowing FDR control as long as the parametric form of the distribution of X is known. A number of publications have explored the construction of knockoffs with approximated distributions of X. For instance, Romano et al. (2020) developed a Deep knockoff machine using deep generative models, Liu and Zheng (2019) created a Model-X generating method employing deep latent variable models, and more recently, Bates et al. (2021) proposed an efficient general metropolized knockoff sampler. Spector and Janson (2022) suggested constructing knockoffs by minimizing the reconstructability of features. Knockoff-based methods have also been extended to test the intersection of null hypotheses, leading to the development of the group and multitask knockoff methods (Dai and Barber, 2016) and prototype group knockoff methods (Chen et al., 2019).

#### Current Advance in FDR Control for Identifying Simultaneous Signals

Simultaneous signal detection has been explored using BH procedure-based methods (Heller et al., 2014; Bogomolov and Heller, 2013, 2018), local FDR (Chi, 2008; Heller and Yekutieli, 2014), and nonparametric approaches (Zhao and Nguyen, 2020). These methods rely on the independence, or positive regression dependency property of the features, which do not hold for most metabolomics studies. Recently, Li et al. (2021) and Dai and Zheng (2023) introduced the multi-environment knockoff and the simultaneous knockoff methods for feature selection and identifying consistent associations, potentially useful for detecting mutual BC and CRC risk factors. However, all these methods do not allow the existence of missing data or measurement errors, which presents an important and unavoidable issue in metabolomic data analyses.

### 1.2 Our Contributions

In this paper, we evaluated the performance of different knockoff extension methods to handle missing values and/or measurement errors in the context of FDR control for variable selection. In addition, we propose a method to select mutual metabolomics predictors for not only one, but multiple clinical outcomes. The main contributions of this paper are summarized below:

1. We construct a knockoff-based procedure for FDR-controlled multiple testing when there are measurement errors and/or missing data in predictors. This procedure can work on general conditional dependence models Y∣X and data structures in X. It can also identify mutual signals for multiple outcomes (e.g. BC and CRC).
2. We demonstrate the FDR control performance and the power of our method with extensive simulation settings. We also illustrate the application with the Women’s Health Initiative (WHI) data examples.

The rest of the paper is organized as follows. In Section 2, we present notations and details of our proposed variable selection framework to control FDR when there are missing data and measurement errors in the predictors. The method can also identify mutual signals for multiple outcomes. In Section 3, we show the empirical performance of the proposed method under different model assumptions and data structures. Finally, in Section 4, we apply the proposed method to a nested case-control study of BC and CRC among WHI Bone Mineral Density (BMD) Subcohort data.

## 2 Methods

### 2.1 Notations

For any positive integer N, denote [N]={1,…,N}. For the n data samples without missingness and measurement error, we assume they are sampled from the underlying distribution of (Y,X) with Y∈R being the response variable and $\text{X}\in R^{p}$ being the p-dimensional predictor. The samples $\left(Y_{i},X_{i1},\ldots ,X_{ip}\right)\overset{\text{iid}}{\sim }𝒟$, for i∈[n]. We work on the multiple testing problem on the null hypotheses $H_{0j}≔Y⫫X_{j}|{\text{X}}_{-j}$, where ${\text{X}}_{-j}≔\left\{X_{k}:k\in [p]\;\text{and}\;k\neq j\right\}$. We aim at developing a selection procedure returning a selection set $\hat{𝒮}\subseteq [p]$ with a controlled FDR:

(1) $$\text{FDR}\left(\hat{𝒮}\right)=E\left[\text{FDP}\left(\hat{𝒮}\right)\right]=E\left[\frac{\left|\hat{𝒮}\cap ℋ\right|}{\left|\hat{𝒮}\right|\vee 1}\right],$$

where $ℋ=\left\{j\in [p]:H_{0j}\;\text{is}\;\text{true}\right\}$ and ∨ means taking the maximum of the two elements.

Given the potential measurement error, we assume X (the true serum/urine metabolites’ level) is not available and an error-prone version $\text{W}\in R^{p}$ (measured metabolites that subject to various sources of measurement error) is available, where $\text{W}=\text{X}+\epsilon_{w}$ with $\epsilon_{w}\overset{\text{iid}}{\sim }{ℱ}_{w}$ where ${ℱ}_{w}$ can be estimated from external data sources. Typically, we can assume a multivariate normal distribution for $\epsilon_{w}$, i.e., $\epsilon_{w}\sim 𝒩\left(0,{𝚺}_{\epsilon }\right)$. Also, for the potential missing data, we further introduce indicator variables $\Delta \in \{0,1{\}}^{p}$ to indicate the missing mechanism. We let $\Delta_{j}=1$ to indicate that $W_{j}$ is observable and we adopt the missing at random (MAR) assumption, i.e., $P\left(\Delta_{j}=\right.1|\text{W},\text{X})=P\left(\Delta_{j}=1|{\text{W}}_{-j}\right)$ where ${\text{W}}_{-j}≔\left\{W_{k}:k\in [p]\;\text{and}\;k\neq j\right\}$. Notice that this assumption is slightly stronger than the usual assumption of $\text{MAR}\left(P\left(\Delta_{j}=1|\text{W},\text{X}\right)=P\left(\Delta_{j}=1|{\text{X}}_{-j}\right)\right)$ based on true variables due to the potential measurement error. In metabolomic data, this assumption is reasonable, since the missingness is mostly related to the signal detected from the other similar metabolite peaks, rather than the true underlying concentration of other metabolites.

For the remaining of the paper, with n observations, our final observed data will be denoted as (Y,𝚫,𝚫⊙W), where ⊙ represents the Hadamard product (i.e., elementwise product). Here $\text{Y}\in R^{n}$ is a vector of responses for the n individuals, $\text{W}\in R^{n\times p}$ denotes the matrix with elements $W_{ij}$ for individual i and predictor $j,𝚫\in \{0,1{\}}^{n\times p}$ denotes the matrix with elements $\Delta_{ij}$ the indicator variable for individual i and predictor j.

### 2.2 Imputation of Missing Data

In general, we generate K imputed datasets, denoted as $\left(\text{Y},{\text{W}}^{k}\right)$, for k∈[K]. When K=1, we consider simple imputation using the mean of the observed values or half of the minimum of the observed values depending on our assumption on whether the missing is random or is due to a detection limit. Also, we can consider multiple imputation methods with K⩾1 where each dataset can be generated using a chained equation approach. Specifically, we first randomly impute the missing values based on the marginal distribution estimated from those individuals with the variable observed, i.e.,

$$W_{ij}^{k}=\Delta_{ij}W_{ij}+\left(1-\Delta_{ij}\right)\frac{\sum_{i}^{n}\Delta_{ij}W_{ij}}{\sum_{i}^{n}\Delta_{ij}}.$$

Then we will update the imputed value iteratively over all j∈[p] that $\sum_{i}\Delta_{ij}<n$. First, we will fit a regression model of $W_{ij}$ on $W_{i,-j}^{k}$ and $Y_{i}$ for those $\Delta_{ij}=1$. Then we will update $W_{ij}^{k}$ for those $\Delta_{ij}=0$ using the predicted value from the model and current $W_{i,-j}^{k},Y_{i}$. Here the regression models can be in the form of generalized linear models (*default*), classification and regression trees (*cart*), or random forest (*rf*). Alternatively, when a large unlabeled subsample exists, another option is to perform multiple imputations excluding the outcome Y from the above steps. We explore both options numerically in Section 3.

### 2.3 Knockoff Construction

For each imputed dataset ${\text{W}}^{k}$, we construct the knockoff ${\tilde{\text{W}}}^{k}$ using second-order Model-X knockoff by sampling ${\tilde{\text{W}}}^{k}$ from $𝒩(\tilde{\mu },\tilde{𝚺})$, where $\tilde{\mu }={\text{W}}^{k}-{\text{W}}^{k}{𝚺}^{-1}\;\text{diag}\left\{\text{s}\right\},\;\tilde{𝚺}=2\;\text{diag}\{\text{s}\}-\text{diag}\{\text{s}\}{𝚺}^{-1}\;\text{diag}\{\text{s}\}$ and 𝚺 is the variance-covariance matrix of W, such that

$$Cov\left(\left[{\text{W}}^{k},\tilde{{\text{W}}^{k}}\right]\right)=\left(\begin{array}{cc} 𝚺 & 𝚺-\text{diag}\{\text{s}\}\\ 𝚺-\text{diag}\{\text{s}\} & 𝚺 \end{array}\right).$$

where s satisfies $\tilde{𝚺}=2\;\text{diag}\{\text{s}\}-\text{diag}\{\text{s}\}{𝚺}^{-1}\;\text{diag}\{\text{s}\}$ is semi-positive definite and s can be solved using the approximate semidefinite program (ASDP) algorithm as given in Candès et al. (2018). We use the R function *create.second* within the R package *knockoff* to implement this construction method. As a remark, the second order knockoff method works primarily for normally distributed data, and the multivariate Gaussian approximation is reasonable after the log-transformation of the metabolite data; the method is also robust against mild model mis-specifications (Candès et al., 2018).

### 2.4 Test Statistics

One advantage of the Knockoff procedure we adopt for FDR control is that we do not need to know the null distribution of our test statistics, as long as they are compatible with the Knockoff method. We consider a variety of test statistics:

- **Lasso:** We assume a working model in a generalized linear model (GLM) framework
$$f_{Y}\left(y;\theta ,\phi \right)=\text{exp}\left\{\frac{y\theta -b\left(\theta \right)}{a\left(\phi \right)}+c\left(y,\phi \right)\right\},$$
  where $\theta ={\text{X}}^{⊤}\beta$. Here β is the parameter of interest and ϕ is the dispersion parameter while a(⋅),b(⋅),c(⋅) are prespecified functions. The expected response is given by the mean function $\mu (\theta )=b\prime (\theta )=g^{-1}(\theta )$, where $g^{-1}(\cdot )$ is the inverse of a canonical link function g(⋅). We choose the Lasso variable selection procedure and construct the statistics as
$$\hat{\beta }(\lambda )=\text{arg}\;\underset{\beta \in R^{2p}}{\text{min}}\sum_{i=1}^{n}\frac{{\left(Y_{i}-\mu \left({\left[{\text{W}}_{i}{\tilde{\text{W}}}_{i}\right]}^{⊤}\beta \right)\right)}^{2}}{V_{i}}+\lambda \|\beta {\|}_{1},$$
  where $V_{i}=V\left(g^{-1}\left({\left[{\text{W}}_{i}{\tilde{\text{W}}}_{i}\right]}^{⊤}\beta \right)\right)$ and V(⋅) is the variance function specified for the GLM for Y. Then we use the absolute value $\left|{\hat{\beta }}_{j}(\lambda )\right|$ as defined above with a specific λ value or λ selected from cross-validation as test statistics (i.e., $Z_{j}=\left|{\hat{\beta }}_{j}(\lambda )\right|$ and ${\tilde{Z}}_{j}=\left|{\hat{\beta }}_{p+j}(\lambda )\right|$ for j∈[p]).
- **Lasso Order:** We assume the same GLM model and run over a range of λ values decreasing from +∞ (a fully sparse model) to 0 (a fully dense model) and define $Z_{j}\left({\tilde{Z}}_{j}\right)$ as the maximum λ such that ${\hat{\beta }}_{j}(\lambda )\neq 0\left({\hat{\beta }}_{p+j}(\lambda )\neq 0\right)$. If there is no λ such that ${\hat{\beta }}_{j}(\lambda )\neq 0\left({\hat{\beta }}_{p+j}(\lambda )\neq 0\right)$, then we will simply define $Z_{j}\left({\tilde{Z}}_{j}\right)$ as 0.
- **Random Forest (RF):** We use the variable importance factors from the random forest fitting of Y on $\left[\begin{array}{ll} \text{W} & \tilde{\text{W}} \end{array}\right]$ with either fixed tuning parameters or tuning parameters selected from cross-validation as $\left[\begin{array}{ll} \text{Z} & \tilde{\text{Z}} \end{array}\right]$.
- **Generalized Dantzig Selector (GDS):** We choose the GDS variable selection procedure and construct the statistics as ${\hat{\beta }}_{\text{DS}}(\lambda )$, where
$${\hat{\beta }}_{\text{DS}}(\lambda )=\underset{\beta \in R^{2p}}{\text{argmin}}\left[\|\beta {\|}_{1}:\underset{1⩽j⩽p}{\text{max}}\left|\frac{1}{n}\sum_{i=1}^{n}W_{ij}\left\{Y_{i}-\mu \left({\left[{\text{W}}_{i}{\tilde{\text{W}}}_{i}\right]}^{⊤}\beta \right)\right\}\right|⩽\lambda \;\text{and}\;\underset{1⩽j⩽p}{\text{max}}\left|\frac{1}{n}\sum_{i=1}^{n}{\tilde{\text{W}}}_{ij}\left\{Y_{i}-\mu \left({\left[{\text{W}}_{i}{\tilde{\text{W}}}_{i}\right]}^{⊤}\beta \right)\right\}\right|⩽\lambda \right].$$
- **Generalized Matrix Uncertainty Selector (GMUS):** The test statistics is a feasible solution of
$${\hat{\beta }}_{\text{MU}}(\lambda )=\underset{\beta \in R^{2p}}{\text{argmin}}\left\{\|\beta {\|}_{1}:\frac{1}{n}{\left\|\sum_{i=1}^{n}{\left[{\text{W}}_{i}{\tilde{\text{W}}}_{i}\right]}^{⊤}\left\{Y_{i}-\mu \left({\left[{\text{W}}_{i}{\tilde{\text{W}}}_{i}\right]}^{⊤}\beta \right)\right\}\right\|}_{\infty }⩽\lambda +\delta \|\beta {\|}_{1}\right\}.$$
- **Corrected Lasso:** The test statistics can be defined as minimizing the loss
$${\hat{\beta }}_{\text{RCL}}(d)=\underset{\beta :\|\beta {\|}_{1}⩽d}{\text{arg}\;\text{min}}\left\{\sum_{i=1}^{n}\frac{{\left(Y_{i}-\mu \left({\left[{\text{W}}_{i}{\tilde{\text{W}}}_{i}\right]}^{⊤}\beta \right)\right)}^{2}}{V_{i}}-\beta^{⊤}{𝚺}_{\epsilon }\beta \right\},$$
  where ${𝚺}_{\epsilon }$ is the variance-covariance matrix for the measurement error in W, and d can be a pre-fixed tuning parameter or selected from cross-validation.

As a remark, when measurement errors exist, the Corrected Lasso and GMUS methods are known to handle measurement errors appropriately for coefficient estimation and thus the knockoff approach can be applied to control the FDR. The performance of other test statistics is expected to be affected by the measurement errors. The impact of the scales and correlations of measurement errors will be evaluated numerically (see Section 3).

### 2.5 Variable Selection

We first compute the p-value for each feature based on the formula $p_{j}=\frac{1+\sum_{k=1}^{K}1\left\{Z_{j}^{k}⩽{\tilde{Z}}_{j}^{k}\right\}}{1+K}$ that combine the information from different imputed datasets. Here 1{⋅} is the indicator function which takes value 1 if the event holds and 0 otherwise. Then we reorder the feature indices j∈[p] with the decreasing order of ${\text{max}}_{k=1}^{K}\text{max}\left\{\left|Z_{j}^{k}\right|,\left|{\tilde{Z}}_{j}^{k}\right|\right\}$ and we denote the new index of the original feature j as ζ(j). We apply the Selective SeqStep and Selective SeqStep+ procedures (Barber and Candès, 2015) with p-value threshold 0.5 to find the selection threshold ${\hat{k}}_{c}$ such that

$${\hat{k}}_{c}=\text{max}\left\{j\in [p]:\frac{c+\sum_{k=1}^{j}1\left\{p_{\zeta^{-1}(k)}>1/2\right\}}{\sum_{k=1}^{j}1\left\{p_{\zeta^{-1}(k)}⩽1/2\right\}\vee 1}⩽q\right\},$$

for c=0 (SeqStep), 1 (SeqStep+), where q is the FDR level to be controlled at. Then the final selection sets will be ${\hat{S}}_{c}=\left\{j:p_{j}<1/2\right\}\cap \left\{j:\zeta (j)\in \left[{\hat{k}}_{c}\right]\right\}$.

Notice that when the imputation model is correct, and there is no measurement error, i.e., W=X, then we have the imputed datasets $\left(\text{Y},{\text{W}}^{k}\right)\sim 𝒟$ for each k, thus for our knockoff construction and test statistics, it has been shown that $P\left({\text{Z}}_{j}^{k}<{\tilde{\text{Z}}}_{j}^{k}\right)=0.5$ for j∈ℋ (Barber and Candès, 2015; Candès et al., 2018). Since $p_{j}$ is symmetrically distributed and $E\left[p_{j}\right]=\frac{1+K/2}{1+K}=\frac{1}{2}+\frac{1}{2(1+K)}>\frac{1}{2}$ for j∈ℋ, therefore the estimated FDP based on the threshold ${\overset{ˆ}{k}}_{c}$, i.e., $\hat{\text{FDP}}=\frac{c+\sum_{k=1}^{{\overset{ˆ}{k}}_{c}}1\left\{p_{\zeta^{-1}(k)}>1/2\right\}}{\sum_{k=1}^{{\overset{ˆ}{k}}_{c}}1\left\{p_{\zeta^{-1}(k)}⩽1/2\right\}\vee 1}$ is a conservative estimate for the FDP and thus the Selective SeqStep+ procedure controls FDR (Barber and Candès, 2015). Although the increasing number of imputed dataset K could lead to better $p_{j}$ estimations, in practice, we don’t need very large K since we only need to know whether $p_{j}$ is below 0.5 or not and the accurate $p_{j}$ is not necessary when it is far from 0.5. In practice, due to the potential model misspecification and finite sample performance, the imputed distribution might be slightly different from the true distribution, nonetheless, the robustness of the knockoff approach (Barber et al., 2020) ensures that the FDR inflation is small.

### 2.6 Joint Selection for Multiple Outcomes

Now we consider the mutual signal identification problem. Assume we have data from M independent experiments and denote [M]={1,…,M}. Within the m-th experiment, the underlying complete data without measurement errors are $\left(Y_{i}^{m},X_{i1}^{m},\ldots ,X_{ip}^{m}\right)\overset{\text{iid}}{\sim }{𝒟}_{m},i=1,\ldots ,n_{m}$. In our setting, the outcome variables $Y^{1},Y^{2}$ represent the two different cancer outcomes BC and CRC. Define $H_{0j}^{m}$ as the null hypothesis indicating the j-th feature not being a signal in the m-th experiment (i.e. $X_{j}^{m}⫫Y^{m}|{\text{X}}_{-j}^{m}$ where ${\text{X}}_{-j}^{m}≔\left\{X_{k}^{m}:k\in [p]\;\text{and}\;k\neq j\right\}$), and denote ${ℋ}^{m}=\left\{j\in [p]:H_{0j}^{m}\;\text{is}\;\text{true}\right\}$, where [p]≔{1,…,p}. Instead of testing the $H_{0j}^{m}’s$, we are interested in testing the union null hypotheses $H_{0j}=\overset{M}{\underset{m=1}{⋃}}H_{0j}^{m}$, for j∈[p].

We define

$$𝒮=\left\{j\in [p]:H_{0j}\;\text{is}\;\text{false}\right\}\;\text{and}\;ℋ={𝒮}^{c}=\overset{M}{\underset{m=1}{⋃}}{ℋ}^{m}=\left\{j\in [p]:H_{0j}\;\text{is}\;\text{true}\right\}.$$

We aim at developing a selection procedure returning a selection set $\hat{𝒮}\subseteq [p]$ with a controlled FDR, as defined in (1). For this task, we can get test statistics $Z_{j}^{km}$ and ${\tilde{Z}}_{j}^{km}$ for each m separately first as the above sections and then compute the p-values as

$$p_{j}=\frac{1+\sum_{k=1}^{K}1\left\{\prod_{m=1}^{M}\left(Z_{j}^{km}-{\tilde{Z}}_{j}^{km}\right)⩽0\right\}}{1+K}.$$

Then we can use this new p value to get ${\hat{k}}_{c}$ and ${\hat{S}}_{c}$ reordering the feature index by the decreasing order of ${\text{max}}_{k=1}^{K}\prod_{m=1}^{M}\left|Z_{j}^{km}-{\tilde{Z}}_{j}^{km}\right|$ or $\sum_{k=1}^{K}\prod_{m=1}^{M}\left|Z_{j}^{km}-{\tilde{Z}}_{j}^{km}\right|$. This approach is valid under a similar argument as in Dai and Zheng (2023).

## 3 Simulation

In this section, we perform extensive numerical experiments to understand the finite sample performance of the proposed methods in Section Methods 2.

### 3.1 Simulation for Gaussian Distributions

We first show the performance of proposed methods in Section Methods 2 when predictors are sampled from multivariate Gaussian distributions.

#### 3.1.1 Data Generation and Settings

We generate data with various sample sizes n and dimension p. We sample the underlying feature matrix $\text{X}\in R^{n\times p}$ with various variation and correlation settings; and the outcome $\text{Y}\in R^{n}$ from a sparse logistic regression with varied effect sizes. Then we sample the measurement errors $\epsilon_{w}$ and construct features with measurement errors $\text{W}=\text{X}+\epsilon_{w}$ with different measurement error scales and correlations. Next, we sample the missing data indicators 𝚫 under the missing at random (MAR) mechanism with varied missing proportion $p_{\text{mis}}$. Under MAR assumption, we consider both missing probabilities dependent on error-prone variables W (Type W) and the error-free variables X (Type X).

We run 200 simulations under each of the following three different settings:

1. **Data with only missing data but not measurement errors.** Under this setting, we compare the performance of the following methods: Lasso, Lasso Order, and RF with multiple imputations (K=5 imputed datasets). We considered either including or excluding the outcome Y (Imp Y) when performing the imputations and considered three different imputation methods (Imp M): R package MICE with default method (*default*), classification and regression tree (*cart*), and random forest (*rf*).
2. **Data with only measurement errors but not missing data.** We compare the performance of our method with the following statistics as described in Section Test statistics 2.4: Lasso, Lasso Order, RF, GDS, GMUS, and Corrected Lasso.
3. **Data with both missing data and measurement errors.** We compare the performance of our method with the following statistics as described in Section Test statistics 2.4: Lasso, Lasso Order, RF, GDS, GMUS, and Corrected Lasso with multiple imputations.

More details on the data generation and simulation settings are postponed in Web Appendix A.1.

#### 3.1.2 Results

For Setting 1 with only missing data, we compare the performance of three variable selection methods: Lasso, Lasso Order, and RF (Table 1). Across the variety of settings we experimented with, all three variable selection methods effectively control the FDR empirically. The Lasso variable selection method demonstrates the highest power among the tested methods, followed by Lasso Order, and finally, RF. The RF method tends to be conservative in selecting variables, resulting in slightly lower power, which may be attributed to the Lasso method possessing the correctly specified model. The prediction performance of the RF method is satisfactory (Table S1). As the sample size (n) decreases and the number of variables (p) increases, all methods maintain satisfactory FDR control, albeit with a slight reduction in power, as anticipated. The proportion of missing data does not significantly impact the performance of these methods in terms of FDR and power. When examining the three imputation methods, their performances are found to be relatively similar. For smaller sample sizes (n=400), the *default* method offers marginally better power, while the *rf* method slightly outperforms the others for larger sample sizes. The decision to include the dependent variable (Y) in the imputation model does not substantially alter the performance of these methods.

In Table 2, we present the FDP and power for our experiments conducted on data with measurement errors. We compare various variable selection methods, including Lasso, Lasso Order, RF, GDS, Corrected Lasso, and GMUS. Notably, the Corrected Lasso and GMUS methods are specifically designed for measurement error correction. The results are organized according to the number of variables p, effect size, and scale. For FDP, Lasso, Lasso Order, RF, and GDS methods exhibit similar values across all scenarios. When p is small and the effect size is large, the FDP is marginally higher than the nominal FDR. Corrected Lasso consistently displays lower FDP values than the nominal FDR, but it tends to be slightly over-conservative. The GMUS method generally yields FDP values comparable to those of Lasso and Lasso Order. Regarding power, the Lasso method persistently achieves higher values compared to other methods across all settings. The GDS and Lasso Order methods also demonstrate satisfactory power, while the RF method consistently exhibits lower power. The GMUS method’s power performance is comparable to that of Lasso Order and GDS, while Corrected Lasso consistently shows the lowest power values among all methods. Overall, as the number of variables increases, FDP experiences a slight decrease and power undergoes a more noticeable reduction. The effect size has a more pronounced impact on power, with larger effect sizes resulting in higher power values. The scale also affects power, with smaller scales generally leading to increased power values. However, the FDP values remain relatively stable irrespective of changes in effect size or scale.

Table 3 summarizes the FDR and power of our proposed methods under simulation settings with both measurement errors and missing data. The variable selection methods we included are the same as in Setting 2. We set the sample size n=1000 and consider two settings for the dimension P: a low-dimensional setting with p=60 and a high-dimension setting with p=210. For the measurement errors, we consider two levels of Scales: $\sigma_{\epsilon }^{2}=0.1\;\text{or}\;0.6$. For imputation methods, we consider the default, cart, and rf. We compare the performance based on whether to include outcome Y in the imputation model. The variable selection methods Lasso, Lasso Order, RF, and GDS do not consider measurement errors, while Corrected Lasso and GMUS correct measurement errors. Here, we present the result in Table 3 where the missing probabilities depend on W rather than X with smaller scales. The additional simulation results can be found in Web Appendix A.2. Tables S2 and S3.

With the small scale of measurement errors (Scale = 0.1), the FDRs for most methods are controlled close to or below the nominal value of 0.2, with Corrected Lasso consistently having the lowest FDR values across all conditions. The results are observed for both low and high dimensions, Type W and Type X, and for different Imp M and Imp Y conditions. With bigger scale measurement errors (Scale = 0.6), for Type W, the FDR is under control for most of the methods except Lasso Order and RF, while for Type X, only the RF and corrected Lasso method controls FDR while the other methods fail. In terms of power, all methods show a decrease as the dimension increases. When the measurement error is small, Lasso, GDS, and GMUS maintain a high power of 0.9, whereas Lasso Order and RF experience a significant decline in power. Corrected Lasso has relatively low power across all settings. When the measurement error is larger, all methods show a substantial power reduction. Overall, Lasso, GDS and GMUS achieve the best performance in terms of power, followed by Lasso Order and RF, while Corrected Lasso consistently exhibits poor power. To demonstrate the performance gain with our proposed methods to work with missing data and measurement errors for Setting 3, we further compared our methods with directly applying Lasso with knockoff (targeted q=0.2) to the dataset, and using minimal value imputation to treat the missing data (Oracle). The Oracle method has satisfactory power but fails in controlling the FDR. With $\sigma_{\epsilon }^{2}=0.1$, this Oracle method has power = 0.95 and FDP = 0.33; with $\sigma_{\epsilon }^{2}=0.6$, this Oracle method has power = 0.87 and FDP = 0.34. More details on this comparison can be found in Web Appendix A.2.

In Table S5 of Web Appendix A.3, we show the performance of proposed methods in Settings 2 and 3 for detecting mutual signals from 2 datasets. The simultaneous knockoff method with our procedure to handle missing data and measurement errors (GMUS) still controls the FDR and achieves comparable power.

### 3.2 Simulation from Empirical Data Distributions

In this section, we perform numerical experiments based on the real metabolomics data from the LC-MS platform (RelQuant).

#### 3.2.1 Data generation and settings

We generate data with the same sample size n=1331 and dimension p=148 as the real data in the LC-MS platform (RelQuant). The data contains both missing values and measurement errors. We approximate the distribution of W from the empirical distribution of real data (details can be found in Web Appendix A.4).

The outcome Y is a binary outcome from a logistic regression. The missing data indicators R are also sampled under the missing at random (MAR) mechanism with proportion $p_{\text{mis}}=0.15$. The same with the Gaussian data simulation (setting for data with both missing data and measurement errors), we compare the performance of the following methods: Lasso, Lasso Order, RF, GDS, GMUS, and Corrected Lasso with multiple imputations.

We run 200 simulations under each of the settings. More details on the data generation and simulation settings are postponed in Web Appendix A.4.

#### 3.2.2 Results

Table 4 illustrates the FDR and power under the empirical real data conditions, including missing values and measurement errors, for scenarios where missing probabilities depend on W (Type W) rather than X (Type X). Additional simulation results, focusing on missing probabilities dependent on X, are available in Web Appendix A.4. We also examine two levels of measurement error scales $\left(\sigma_{\epsilon }^{2}\right)$, and consider the three imputation methods (Imp M): *default*, *cart*, and *rf*, as well as evaluate the impact of imputing Y (Imp Y) on performance.

In terms of FDR, with small measurement errors (scale = 0.1), Lasso, Lasso Order, RF, Corrected Lasso, and GMUS effectively controlled the FDR, whereas GDS do not. At a larger error scale (scale = 0.5), only RF and Corrected Lasso strictly maintain the FDR below 0.2. GMUS managed to keep the FDR around 0.2, but Lasso, Lasso Order and GDS were unsuccessful in controlling the FDR. Regarding power, Lasso, GDS, and GMUS exhibit superior performance, followed by RF. However, Corrected Lasso demonstrates the limited power. These results were consistent under various imputation conditions (Imp M and Imp Y).

A comparative analysis of the performance of six variable selection methods between Gaussian distributed data and empirical data distribution from the LC-MS platform reveals inconsistencies in the Lasso method performance. Specifically, Lasso effectively controls FDR in some conditions (Type W) for Gaussian data but fails to do so for empirical data distributions. Different imputation methods (Imp M) and whether considering outcome variable Y (Imp Y) during the imputation does not impact the performance significantly. Overall, when missing values but no measurement error exist in the data, the Lasso and Lasso order methods perform the best; when measurement errors exist in the data, the GMUS method performs the best with the highest power, and the controlled FDR under the assumption that the missing probability depends on the error-prone variables W. In our real data analysis, this missing mechanism assumption is likely to hold, so, GMUS is considered as one of the primary methods in addition to Lasso and Lasso Order for the real data analysis.

## 4 Real Data Analysis

We analyzed serum and urine specimens from the WHI BMD data (181 CRC cases; 577 BC cases; 758 matched controls) using several metabolomics platforms. The details on how the matched samples were selected can be found in Web Appendix B.1. We applied global metabolomics platforms (gas chromatography–mass spectrometry (GC-MS) and nuclear magnetic resonance (NMR)) for profiling urine metabolites and targeted platforms for profiling serum metabolites. In serum, using liquid chromatography with tandem mass spectrometry (LC-MS/MS), we targeted water-soluble metabolites covering over 50 major metabolic pathways, and using the recently developed Lipidyzer platform, we detected about 900 lipids from 13 different classes: Cholesterol ester (CE), Ceramides (CER), Diacylglycerol (DAG), Dihydroceramides (DCER), Free fatty acids (FFA), Hexosylceramides (HCER), Lactosylceramide (LCER), Lysophosphatidylcholine (LPC), Lysophosphatidylethanolamine (LPE), Phosphatid-ylcholine (PC), Phosphatidylethanolamine (PE), Sphingomyelin (SM), Triacylglycerol (TAG). Over 1500 metabolites were obtained from urine and serum samples using these four complementary analytical platforms. The proportion of missing data as well as the signal noise ratio for measurement errors are summarized in Table S7 in Web Appendix B where we can see that the GC-MS and LC-MS suffer most from the measurement error with some SNR less than one and GC-MS has the most missing data. We applied the knockoff method with missing and measurement errors to find the metabolite factors associated with BC, CRC, and shared factors for both cancers using the proposed methods.

### 4.1 Data Preprocessing

We first preprocessed the data from the four different platforms (NMR, GC-MS, LC-MS, and lipidomic) to remove outliers, batch effects, and variables with excessive missing values (details see Web Appendix B.2). For LC-MS and lipidomic data, we considered both concentration and composition data which led to a total of 6 groups of metabolites and we analyze each group separately. The binary variable case/control of certain cancers served as our response variable. Here the preprocessed metabolites in non-quality control (QC) samples form the predictor matrix $\text{W}\in R^{n\times p}$, where n is the number of patients and p is the number of metabolites.

To achieve the goals of finding the risk metabolite factors for BC and CRC, we analyzed the data including the BC cases vs all controls, and the CRC cases vs all controls respectively. Then the summary statistics were combined using the method described in Section Joint selection for multiple outcomes to identify the shared factors associated with both breast and CRC.

To build the model, first, for each platform dataset, we imputed the data by different imputation methods including half-min imputation, and multiple imputations with predictive mean matching method. For the multiple imputations, we performed MICE with (K=5) using different analytic data for each outcome (i.e., BC cases + all controls for BC analysis and CRC + all controls for CRC analysis) were used to impute the missing values, and outcomes were included in the multiple imputation procedure. The variance-covariance matrix for the measurement errors was estimated using QC samples (details can be found in Web Appendix B.3). Then, we applied three preferred methods (Lasso, Lasso Order, GMUS) and three alternative methods (RF, GDS, Corrected Lasso) as described in Section Methods to generate test statistics. Then we applied our knockoff and simultaneous knockoff variable selection procedures with a target FDR level of 0.1. To make our results more robust, we performed stability selection by running different methods 100 times and recording the percentage of the replications each variable was selected.

For sensitivity analysis, we performed the same analysis as above but using different analytical data for each outcome (i.e., BC cases + matched BC control for BC analysis and CRC cases + matched CRC control for CRC analysis).

### 4.2 Results

We presented the metabolites that were selected to be associated with BC, CRC, and both of these two cancers with ⩾ 50% of the replications in Tables 5, 6 and 7, respectively when using the three preferred variable selection methods (i.e., Lasso, Lasso Order, and GMUS). The directions of the marginal associations are also indicated (+: positive, −: negative). The list of the metabolites that were selected to be associated with BC, and CRC with ⩾ 10% of the replications using all methods can be found in Web Appendix C.

Comparing the three variable selection methods, Lasso Order gives the most selections, followed by Lasso and GMUS. Across different methods, there are metabolites that are mutually selected by the different methods, for example, TAG 48:5(FA 18:3) and DAG 14:1/18:1; on the other hand, each method also selects some unique metabolites. Comparing the two imputation options, they produce relatively consistent results. Since we only select variables with high replications (⩾ 50%, with FDR controlled for every replication), we include selections from all the proposed variable selection methods as identified signals. Sensitivity analysis using single cancer-type cases and their own matched controls is performed. More details on the analysis and the selected metabolites are presented in Web Appendix B. The variables selected are largely the same, although fewer variables are selected due to reduced sample sizes.

## 5 Discussion

In conclusion, our extensive empirical studies show that appropriately handling missing data and measurement errors using the knockoff approach can control FDR at the targeted rate and gain power in terms of finding metabolites associated with BC and CRC risks. When the general simultaneous knockoff methods (Dai and Zheng, 2023) are used for two outcomes (see Web Appendix A.3 for details), we find that appropriately handling missing data with multiple imputation and measurement error using GMUS will control FDR for multiple outcomes at the targeted rate.

We identified a group of metabolites that are associated with either BC, CRC, or both cancers. The biomarker findings are largely consistent with the existing literature. For example, pentanedioic acid derivatives have been proposed as a potential agent for the treatment of BC (Zhang et al., 2022). N-methyl nicotinic acid level in LC-ESI-MS has been reported to be positively associated with BC (Valko-Rokytovská et al., 2021). The increase of 3-hydroxybutyric acid (3HBA) level has been found as an indication of the increased fatty acid oxidation, a hallmark for cancer aggressiveness (Cappelletti et al., 2017). For CRC, serum 2,3-dihydroxybutanoic acid has been reported as a biomarker (Loktionov, 2020). Glucose (Vulcan et al., 2017), glycerate (Ni et al., 2014), adenosine (Hata et al., 2023), N-methyl nicotinic acid, cystine (Miller et al., 2013), malate (Neitzel et al., 2020), histidine (Rothwell et al., 2023) and CER (16:0) (Machala et al., 2019) have also been discovered to be associated with CRC in other independent studies. Choline has been reported to be positively associated with risks for both BC (Bae et al., 2014) and CRC (Xu et al., 2008). The results confirm some findings of previous literature and also discover a few new potential metabolite biomarkers for future validation. The matching method (see details in Web Appendix B) to construct the analytical dataset ensures non-overlapping independent samples for the BC and CRC outcomes to satisfy the assumption of the simultaneous knockoff method Dai and Zheng (2023). One small caveat of the case-control study is the potential X distribution shift. However, this impact is very minor in our analysis given the distribution of X is estimated using the case-control study rather than larger population data.

One limitation of the current study is our cohort only contains post menopausal women. A limitation of the current study is the small sample size for QCs which leads to large variations in the estimation of the variance-covariance matrix of measurement error. This could make the measurement error correction method vulnerable to potential misspecification of the measurement error distribution and be sensitive to the result of an outlier in 1 or 2 QC pairs. In the current application, we use second-order Model-X knockoff construction. When the variables X and measurement errors both follow the multivariate Gaussian distribution, the second-order condition is sufficient to guarantee the exchangeability of the whole distribution. However, when the variable is non-Gaussian distributed, the higher order moment mismatching could lead to the difference in the distribution of Z and $\tilde{Z}$ for null variables, which will affect the FDP from the knockoff, especially the simultaneous knockoff procedure. When the measurement error is non-Gaussian, Corrected Lasso will not be suitable, and estimating the optimal error bound for GMUS will be challenging and require a larger sample size for QCs. Further measurement error correction methods for both the estimation of the effect and the variable selection will be worth future research. Another issue is that some metabolites are highly correlated to each other, which will make the knockoff feature very close to the original feature and thus lead to low power. Further method development for group variable selection (by treating metabolites from the same pathway as a group or treating highly correlated metabolites as a group) is worth further exploration but is beyond the scope of this paper. In addition, the current analysis is based on considering each platform’s data separately. In the future, methods need to be developed to handle multiple platform data together by solving the challenge of very different measurement scales and potential screening methods to reduce the number of features to allow powerful knockoff construction.

## Supplementary Material

Supplemental

We provide an additional pdf file that includes additional simulation results and real data analysis. The R codes for the analysis of this paper are available at https://github.com/RunqiuWang22/Variable_Selection_FDR_noisy.

## Figures and Tables

**Table 1:** Simulation results (FDP and Power) for Setting 1 (missing data) varying n,p and $p_{\text{mis}}$; with different imputation methods (Imp M) and choice of whether to include Y in the imputation (Imp Y).

n	p	${\text{p}}_{\text{mis}}$	Imp M	Imp Y	FDP			Power		
					Lasso	Lasso Order	RF	Lasso	Lasso Order	RF
400	60	0.05	cart	yes	0.15	0.14	0.14	0.99	0.65	0.46
400	60	0.05	cart	no	0.16	0.15	0.14	0.98	0.67	0.50
400	60	0.05	default	yes	0.18	0.13	0.15	0.99	0.64	0.50
400	60	0.05	default	no	0.18	0.13	0.16	0.99	0.66	0.52
400	60	0.05	rf	yes	0.17	0.14	0.14	0.99	0.66	0.50
400	60	0.05	rf	no	0.16	0.15	0.13	0.99	0.65	0.49
400	60	0.15	cart	yes	0.17	0.14	0.17	0.98	0.64	0.54
400	60	0.15	cart	no	0.15	0.14	0.13	0.98	0.67	0.47
400	60	0.15	default	yes	0.17	0.15	0.13	0.99	0.68	0.50
400	60	0.15	default	no	0.17	0.17	0.14	0.99	0.70	0.49
400	60	0.15	rf	yes	0.17	0.15	0.15	0.98	0.67	0.50
400	60	0.15	rf	no	0.18	0.17	0.16	0.98	0.70	0.52
1000	60	0.05	cart	yes	0.17	0.18	0.18	1.00	0.94	0.76
1000	60	0.05	cart	no	0.17	0.19	0.15	1.00	0.95	0.72
1000	60	0.05	default	yes	0.20	0.17	0.16	1.00	0.93	0.70
1000	60	0.05	default	no	0.18	0.19	0.16	1.00	0.94	0.73
1000	60	0.05	rf	yes	0.19	0.17	0.17	1.00	0.92	0.74
1000	60	0.05	rf	no	0.18	0.17	0.17	1.00	0.93	0.73
1000	60	0.15	cart	yes	0.18	0.20	0.18	1.00	0.94	0.73
1000	60	0.15	cart	no	0.19	0.20	0.18	1.00	0.94	0.74
1000	60	0.15	default	yes	0.20	0.18	0.16	1.00	0.94	0.73
1000	60	0.15	default	no	0.19	0.18	0.18	1.00	0.93	0.73
1000	60	0.15	rf	yes	0.22	0.22	0.18	1.00	0.94	0.75
1000	60	0.15	rf	no	0.21	0.21	0.18	1.00	0.93	0.74
1000	120	0.05	cart	yes	0.18	0.17	0.17	1.00	0.85	0.58
1000	120	0.05	cart	no	0.18	0.16	0.16	1.00	0.84	0.59
1000	120	0.05	default	yes	0.19	0.15	0.16	1.00	0.84	0.57
1000	120	0.05	default	no	0.19	0.16	0.15	1.00	0.84	0.56
1000	120	0.05	rf	yes	0.18	0.16	0.16	1.00	0.85	0.58
1000	120	0.05	rf	no	0.18	0.17	0.16	1.00	0.86	0.58
1000	120	0.15	cart	yes	0.19	0.18	0.17	1.00	0.85	0.59
1000	120	0.15	cart	no	0.17	0.18	0.17	1.00	0.85	0.58
1000	120	0.15	default	yes	0.20	0.19	0.16	1.00	0.87	0.58
1000	120	0.15	default	no	0.18	0.18	0.18	1.00	0.84	0.60
1000	120	0.15	rf	yes	0.20	0.18	0.19	1.00	0.85	0.62
1000	120	0.15	rf	no	0.19	0.19	0.18	1.00	0.85	0.59

**Table 2:** Simulation results (FDP and Power) for Setting 2 (data with measurement errors) for n=1000, varying p, scales of the effect $\left(A_{\beta }\right)$ and errors $\left(\sigma_{\epsilon }^{2}\right)$.

p	${\text{A}}_{\beta }$	$\sigma_{\epsilon }^{2}$	Lasso	Lasso Order	RF	GDS	Corrected Lasso	GMUS
FDP								
60	0.5	0.6	0.23	0.21	0.22	0.21	0.13	0.21
60	0.5	1	0.23	0.22	0.20	0.21	0.19	0.22
60	1.5	0.6	0.25	0.25	0.24	0.22	0.15	0.22
60	1.5	1	0.25	0.25	0.28	0.23	0.25	0.25
120	0.5	0.6	0.21	0.21	0.17	0.20	0.05	0.20
120	0.5	1	0.18	0.19	0.16	0.18	0.14	0.17
120	1.5	0.6	0.20	0.20	0.17	0.18	0.07	0.17
120	1.5	1	0.20	0.20	0.20	0.20	0.10	0.19
Power								
60	0.5	0.6	0.91	0.84	0.71	0.91	0.44	0.89
60	0.5	1	0.88	0.79	0.61	0.87	0.49	0.83
60	1.5	0.6	0.94	0.88	0.75	0.94	0.44	0.92
60	1.5	1	0.93	0.87	0.75	0.92	0.63	0.90
120	0.5	0.6	0.80	0.72	0.49	0.78	0.17	0.76
120	0.5	1	0.70	0.59	0.40	0.67	0.25	0.64
120	1.5	0.6	0.83	0.77	0.54	0.81	0.15	0.80
120	1.5	1	0.76	0.65	0.49	0.74	0.25	0.72

**Table 3:** Simulation results (FDP and Power) for Setting 3 (data with both measurement errors and missing data) for $n=1000,\sigma_{\epsilon }^{2}=0.1,p_{\text{mis}}=0.15$, and $A_{\beta }=1$, varying p, Imp M and Imp Y when the missing probability depends on the error-prone variables W.

p	Imp M	Imp Y	Lasso	Lasso Order	RF	GDS	Corrected Lasso	GMUS
FDP								
60	default	yes	0.18	0.19	0.15	0.20	0.02	0.15
60	default	no	0.16	0.20	0.18	0.19	0.02	0.13
60	cart	yes	0.16	0.20	0.18	0.19	0.02	0.13
60	cart	no	0.18	0.18	0.17	0.17	0.03	0.12
60	rf	yes	0.17	0.19	0.17	0.19	0.02	0.14
60	rf	no	0.17	0.20	0.15	0.19	0.02	0.14
210	default	yes	0.20	0.15	0.15	0.17	0.00	0.16
210	default	no	0.18	0.16	0.15	0.17	0.00	0.19
210	cart	yes	0.18	0.17	0.16	0.17	0.00	0.17
210	cart	no	0.18	0.17	0.16	0.18	0.00	0.19
210	rf	yes	0.19	0.18	0.17	0.18	0.00	0.18
210	rf	no	0.19	0.18	0.17	0.17	0.00	0.18
Power								
60	default	yes	1.00	0.92	0.80	1.00	0.46	0.99
60	default	no	1.00	0.92	0.79	1.00	0.42	0.98
60	cart	yes	1.00	0.92	0.80	1.00	0.44	0.98
60	cart	no	1.00	0.91	0.80	1.00	0.43	0.98
60	rf	yes	1.00	0.91	0.80	1.00	0.42	0.98
60	rf	no	1.00	0.92	0.78	1.00	0.42	0.98
210	default	yes	0.91	0.63	0.44	0.88	0.000	0.89
210	default	no	0.90	0.66	0.46	0.87	0.000	0.89
210	cart	yes	0.90	0.65	0.45	0.87	0.000	0.88
210	cart	no	0.90	0.65	0.45	0.87	0.001	0.88
210	rf	yes	0.91	0.68	0.48	0.89	0.002	0.88
210	rf	no	0.90	0.67	0.47	0.88	0.000	0.88

**Table 4:** Simulation results (FDP and Power) for Empirical Data Distribution from LC-MS platform (RelQuant) for $n=1331,\;p=148,\;p_{\text{mis}}=0.15$, and $A_{\beta }=1$, varying $\sigma_{\epsilon }^{2}$, Imp M and Imp Y when the missing probability depends on the error-prone variables W.

$\sigma_{\epsilon }^{2}$	Imp M	Imp Y	Lasso	Lasso Order	RF	GDS	Corrected Lasso	GMUS
FDP								
0.1	default	yes	0.20	0.19	0.13	0.21	0.04	0.19
0.1	default	no	0.18	0.19	0.14	0.20	0.04	0.18
0.1	cart	yes	0.18	0.18	0.14	0.20	0.04	0.19
0.1	cart	no	0.18	0.18	0.15	0.20	0.05	0.19
0.1	rf	yes	0.19	0.19	0.14	0.21	0.04	0.20
0.1	rf	no	0.19	0.20	0.13	0.22	0.03	0.20
0.5	default	yes	0.24	0.23	0.14	0.23	0.07	0.19
0.5	default	no	0.23	0.22	0.14	0.22	0.06	0.19
0.5	cart	yes	0.21	0.20	0.14	0.23	0.07	0.20
0.5	cart	no	0.22	0.22	0.14	0.22	0.07	0.20
0.5	rf	yes	0.23	0.24	0.16	0.22	0.07	0.21
0.5	rf	no	0.22	0.24	0.15	0.23	0.07	0.21
Power								
0.1	default	yes	1.00	0.94	0.71	1.00	0.16	1.00
0.1	default	no	1.00	0.93	0.72	1.00	0.14	1.00
0.1	cart	yes	1.00	0.93	0.71	1.00	0.14	1.00
0.1	cart	no	1.00	0.93	0.71	1.00	0.15	1.00
0.1	rf	yes	1.00	0.93	0.71	1.00	0.14	1.00
0.1	rf	no	1.00	0.93	0.71	1.00	0.16	1.00
0.5	default	yes	1.00	0.94	0.71	1.00	0.29	0.99
0.5	default	no	1.00	0.93	0.69	0.99	0.29	0.99
0.5	cart	yes	0.99	0.92	0.70	0.99	0.32	0.99
0.5	cart	no	1.00	0.93	0.70	0.99	0.29	0.99
0.5	rf	yes	0.99	0.94	0.71	0.99	0.33	0.99
0.5	rf	no	0.99	0.94	0.71	0.99	0.31	0.99

**Table 5:** Metabolites that are robustly (selected among ⩾50% of replications) associated with BC risks and the direction of their marginal association to the BC risks.

Method	Platform	Half Min Imputation	Multiple Imputation
Lasso	GC-MS	Alpha-ketoglutarate (76%)(−)	
Lasso	Lipidyzer (composition)		TAG 48:5(FA18:3) (80%)(−) DAG 14:1/18:1 (78%)(+)
Lasso	Lipidyzer (concentration)	DAG 14:1/18:1 (97%)(+)	DAG 14:1/18:1 (97%)(+) TAG 48:5(FA18:3) (64%)(−)
Lasso Order	NMR	N-methylnicotinic acid (59%)(+)	N-methylnicotinic acid (63%)(+)
Lasso Order	GC-MS	Alpha-ketoglutarate (54%)(−)	
Lasso Order	LC-MS (AbsQuant)	3HBA (67%)(+) Cystine (66%)(−)	
Lasso Order	Lipidyzer (composition)	TAG 47:0(FA15:0) (68%)(−)	TAG 48:5(FA18:3) (80%)(−) DAG 14:1/18:1 (76%)(+)
GMUS	Lipidyzer (composition)	TAG 52:2(FA18:2) (66%)(−)	PC 16:0/18:2 (53%)(+)
GMUS	Lipidyzer (concentration)	DAG 14:1/18:1 (63%)(+)	DAG 14:1/18:1 (93%)(+)
Corrected Lasso	GC-MS	Alpha-ketoglutarate (55%)(−)	Alpha-ketoglutarate (73%)(−)

**Table 6:** Metabolites that are robustly (selected among ⩾50% of replications) associated with CRC risks and the direction of their marginal association to the CRC risks.

Method	Platform	Half Min Imputation	Multiple Imputation
Lasso	GC-MS		2,3-Dihydroxybutanoic acid (93%)(+)
Lasso	LC-MS (AbsQuant)	Glucose (84%)(+) Cystine (52%)(−)	Glucose (62%)(+)
Lasso	LC-MS (RelQuant)	Glycerate (69%)(+) Adenosine (66%)(−)	Adenosine (69%)(−) Glycerate (67%)(+)
Lasso	Lipidyzer (composition)	TAG 48:5(FA18:2) (59%)(+)	
Lasso Order	NMR	N-methylnicotinic acid (76%)(−)	N-methylnicotinic acid (54%)(−)
Lasso Order	GC-MS		2,3-Dihydroxybutanoic acid (63%)(+)
Lasso Order	LC-MS (AbsQuant)	Cystine (100%)(−) 3HBA (75%)(+)	Cystine (94%)(−)
Lasso Order	LC-MS (RelQuant)	Malate (57%)(+)	
Lasso Order	Lipidyzer (composition)	TAG 48:5(FA18:2) (60%)(+)	
Lasso Order	Lipidyzer (concentration)	TAG 47:2(FA14:0) (61%)(−)	
GMUS	LC-MS (AbsQuant)		Histidine (69%)(−)
GMUS	Lipidyzer (concentration)	CER 16:0 (52%)(+)	CER 16:0 (66%)(+)

**Table 7:** Metabolites that are robustly (selected among ⩾50% of replications) associated with both BC and CRC risks and the direction of their marginal association to these two cancer risks.

Method	Platform	Half Min Imputation	Multiple Imputation
Lasso Order	NMR		N-methylnicotinic acid (57%)(B:+)(C:−)
Lasso Order	LC-MS (AbsQuant)	Cystine (89%)(B:−)(C:−) 3HBA (68%)(B:+)(C:+)	Cystine (99%)(B:−)(C:−) 3HBA (83%)(B:+)(C:+) Glutamic acid (78%)(B:+)(C:+)
GMUS	LC-MS (AbsQuant)	Choline (56%)(B:+)(C:+)	Choline (63%)(B:+)(C:+)

## Data Availability

The data that support the findings in this paper is not publicly available but could be requested through WHI in a collaborative mode as described on the Women’s Health Initiative website (www.whi.org).
