# Supplementary material for: Variable Selection with FDR Control for Noisy Data – An Application to Screening Metabolites that Are Associated with Breast Cancer and Colorectal Cancer
Source: J Data Sci. Author manuscript; Available in PMC 2026 Apr 25. (PMC13108675; doi:10.6339/25-jds1166)
Supplement: Supplemental [file NIHMS2164524-supplement-Supplemental.pdf]

Journal of Data Science 0 (0), 1–22 DOI: 0000
July 2025 Research Article

Supporting Information for "Variable selection with FDR control for noisy data – an application to screening metabolites that are associated with breast cancer and colorectal cancer"

Runqiu Wang [1], Ran Dai[1,*], Ying Huang[2], Marian L. Neuhouser[2], Johanna W. Lampe[2], Daniel Raftery[3], Fred K. Tabung[4], Cheng Zheng[1,*]

[1]*Department of Biostatistics, University of Nebraska Medical Center, Omaha, Nebraska, U.S.A.*
[2]*Division of Public Health Sciences, Fred Hutchinson Cancer Center, Seattle, Washington, U.S.A.*
[3]*Department of Anesthesiology and Pain Medicine, University of Washington, Seattle, Washington, U.S.A.*
[4]*Department of Internal Medicine, College of Medicine and Comprehensive Cancer Center, The Ohio State University, Columbus, Ohio, U.S.A.*

Appendix A: Additional simulation details, settings and results

A.1: Appendix for Simulation details

Denoting the sample size as n and number of features p, we generate data with the combinations $(n, p) = (1000, 60)$, $(1000, 120)$ or $(400, 60)$.

First sample the feature matrix $\boldsymbol{X} \sim \mathcal{N}(\mathbf{0}, \boldsymbol{\Sigma}_X)$. Here we let $\boldsymbol{\Sigma}_X = AR(\sigma_X, \rho_X, p)$, where $AR(\sigma, \rho, p)$ denotes a $p \times p$ matrix with (i, j)-th element equals to $\sigma^2 \rho^{|i-j|}$. We use σ^2 to control the magnitude of the variation, and ρ to control the correlation between the predictors. Specifically, we set $\sigma_X^2 = 1$ and $\rho_X = 0.5$.

Second, we sample outcome Y_i from a logistic regression model

$$\log \frac{\mathbb{P}(Y_i = 1|\mathbf{X_i})}{\mathbb{P}(Y_i = 0|\mathbf{X_i})} = \beta_0 + A_{\boldsymbol{\beta}} \mathbf{X}_i^\top \boldsymbol{\beta} \text{ for } i \in [n].$$

where $\boldsymbol{\beta} = (\mathbf{1}_{s/3} \otimes (3, 1.5, 0, 0, 2, 0, 0), \mathbf{0}_{p-7s/3}) \odot \epsilon$ is a sparse vector with sparsity level $s = p/4$, $A_{\boldsymbol{\beta}} = 0.5$ or 1.5 controls the magnitude of the effect while ϵ is a vector of independent Rademacher variables, and β_0 controls the prevalence of outcome and is set as $\beta_0 = -1$.

Third, we sample measurement error $\boldsymbol{\epsilon}_w \sim \mathcal{N}(\mathbf{0}, \boldsymbol{\Sigma}_\epsilon)$ and calculate $\boldsymbol{W} = \boldsymbol{X} + \boldsymbol{\epsilon}_w$. Here we consider $\boldsymbol{\Sigma}_\epsilon = AR(\sigma_\epsilon, \rho_\epsilon, p)$ where $\sigma_\epsilon^2 = 0$, 0.1, 0.6 or 1 controls the scale of measurement errors, and ρ_ϵ controls the correlation between measurement errors, which is set at $\rho_\epsilon = 0.3$.

Fourth, we sample missing data indicators Δ_{ij} under the missing at random (MAR) mechanism. We first randomly choose subsets $S_{mis0} \subset [p] \cap \mathcal{H}$ and $S_{mis1} \subset [p] \cap \mathcal{H}^c$ so that $|S_{mis0}| = \pi_{mis} \cdot (p - s)$ and $|S_{mis1}| = \pi_{mis} \cdot s$ where π_{mis} controls the proportion of variables that will contain missing values and is set at $2/15$ to approximate the proportion of variables with more than 5% missing in our real data set or at 0 for setting without missing data. Then for each $j \in S_{mis} = S_{mis0} \cup S_{mis1}$, we sample Δ_{ij} independently from a sequence of logistic regression models

$$\log \frac{\mathbb{P}(\Delta_{ij} = 1|\mathbf{W}_{i,-j})}{\mathbb{P}(\Delta_{ij} = 0|\mathbf{W}_{i,-j})} = \eta_{0j} + \mathbf{W}_{i,-j}^\top \boldsymbol{\eta}_j,$$

*Corresponding author. Email: ran.dai@unmc.edu or cheng.zheng@unmc.edu.

where the intercept η_{0j} is used to control the average proportion of missing at 5% or 15% and elements of $\boldsymbol{\eta}_j$ are independently sampled from Uniform$[-2, 2]$. For $j \notin S_{mis}$, we let $\Delta_{ij} = 1$. Additional simulation when the missing depend on $\mathbf{X}$ rather than $\mathbf{W}$ are also considered where we will then sample Δ_{ij} for each $j \in S_{mis}$ by

$$\log \frac{\mathbb{P}(\Delta_{ij} = 1|\mathbf{X}_{i,-j})}{\mathbb{P}(\Delta_{ij} = 0|\mathbf{X}_{i,-j})} = \eta_{0j} + \mathbf{X}_{i,-j}^{\top}\boldsymbol{\eta}_j,$$

where the intercept η_{0j} is used to control the average proportion of missing among those variables with missing value at $p_{mis} = 5\%$ or 15% and elements of $\boldsymbol{\eta}_j$ are independently sampled from Uniform$[-2, 2]$.

We first consider a setting with $\boldsymbol{\Sigma}_\epsilon = \mathbf{0}$, which is a setting with only missing data but not measurement errors. Under this setting, we compare the performance of the following methods: Lasso, Lasso Order, RF in terms of the FDR control and power, calculated from 200 replicates when using multiple imputations from chained equations with 5 imputed datasets each. We considered either including or excluding the outcome Y when performing the imputations and consider three different imputation methods (*default* method using generalized linear models, classification and regression tree (*cart*), and random forest (*rf*).

The second setting is with $\pi_{mis} = 0$, which reflects a setting with only measurement errors, but not missingness in the data. Under this setting, we compare the performance of the following methods: Lasso, Lasso Order, RF, GDS, GMUS, and Corrected Lasso in terms of FDR control and power calculated from 200 replicates.

The third setting is with $\pi_{mis} \neq 0$ and $\boldsymbol{\Sigma}_\epsilon \neq \mathbf{0}$, which reflects a setting with both missing data and measurement errors. Under this setting, we compare the performance of the following methods: Lasso, Lasso Order, RF, GDS, GMUS, and Corrected Lasso with respect to FDR control and power calculated from 200 replicates when using multiple imputations from chained equations with 5 imputed datasets each.

Appendix A.2: Additional Simulation Settings and Results

To investigate the poor performance in terms of the power of the random forest model, we further evaluate its predictive performance on a test set comprising 20% of the data with the same optimized hyperparameters as Table 1. The classification metrics, including accuracy, precision, recall, F1 score, and Area Under the Curve (AUC), are shown in Table S1. The results demonstrated robust predictive performance of random forest.

The results of simulation settings with both measurement errors and missing data for larger measurement scales where the missing probabilities depend on W are shown in Table S2. The FDR is under control for most of the methods except Lasso Order and RF. Regarding power, the Lasso, GDS, and GMUS methods demonstrated superior performance, while Lasso Order and Random Forest showed moderate effectiveness. The Corrected Lasso method still exhibited notably low power.

Since the missing at-random assumption based on error-prone variables ($\mathbf{W}$) can be strong for some applications, here we present the simulation study to see how the result will be sensitive to that assumption when the truth is that the missing probability depends on the error-free variables ($\mathbf{X}$). The results are summarized in Table S3.

To highlight the performance improvements achieved with our proposed methods for handling missing data and measurement errors, we conducted a comparative analysis under Setting 3. Specifically, we compared our methods against a baseline approach where Lasso with knockoff

filtering was directly applied to the dataset, using a minimal-value imputation method to address missing data (referred to here as the Oracle method). We use the same setting from setting 3, data with both measurement errors and missing data for $n = 1000$, $p = 60$, $p_{mis} = 0.15$, and $A_{\boldsymbol{\beta}} = 1$. We vary the scale of measurement error σ_ϵ^2, and the missing probability depends on the error-prone variables $\mathbf{W}$ or the error-free variables $\mathbf{X}$. The results are shown in Table S4.

Appendix A.3: Additional Simulation Settings and Results for General Simultaneous Knockoff Methods

We generate two independent datasets with the same settings outlined in Table 3, except for the values for the coefficient vectors. First, we sample two feature matrices $\mathbf{X}^1, \mathbf{X}^2$ independently with the same as the description in Appendix A.1. Second, for coefficients, the mutual signals for both datasets are the same, and two non-mutual signals have magnitudes 0.5 and 1 with random direction in both data sets.

$$\begin{aligned}\boldsymbol{\beta}^1 &= (\mathbf{1}_{s/3} \otimes (3, 1.5, 0, 0, 2, 0, 0), \boldsymbol{\omega}_1, \mathbf{0}_2, \mathbf{0}_{p-7s/3-4}) \odot \boldsymbol{\epsilon}, \\ \boldsymbol{\beta}^2 &= (\mathbf{1}_{s/3} \otimes (3, 1.5, 0, 0, 2, 0, 0), \mathbf{0}_2, \boldsymbol{\omega}_2, \mathbf{0}_{p-7s/3-4}) \odot \boldsymbol{\epsilon},\end{aligned}$$

where $\boldsymbol{\omega}_1 = (0.5, 1) \odot \boldsymbol{\epsilon}_1$, $\boldsymbol{\omega}_2 = (0.5, 1) \odot \boldsymbol{\epsilon}_2$, $\boldsymbol{\epsilon}_1, \boldsymbol{\epsilon}_2$, and $\boldsymbol{\epsilon}$ are vectors of independent Rademacher variables. Then Y_i^1, Y_i^2s are generated from logistic regression models

$$\log \frac{\mathbb{P}(Y_i^1 = 1 | \mathbf{X}_i^1)}{\mathbb{P}(Y_i^1 = 0 | \mathbf{X}_i^1)} = \beta_0 + A_{\boldsymbol{\beta}} \mathbf{X}_i^{1\top} \boldsymbol{\beta}^1,$$
$$\log \frac{\mathbb{P}(Y_i^2 = 1 | \mathbf{X}_i^2)}{\mathbb{P}(Y_i^2 = 0 | \mathbf{X}_i^2)} = \beta_0 + A_{\boldsymbol{\beta}} \mathbf{X}_i^{2\top} \boldsymbol{\beta}^2$$

fo $i \in [n]$.where $A_{\boldsymbol{\beta}} = 1$ controls the magnitude of the effect, β_0 controls the prevalence of outcome and is set as $\beta_0 = -1$. The measurement error and missing data indicators are independently sampled for both datasets and the same as the description in Appendix A.1.

We simulate both datasets using Setting 3 (both data with both missing data and measurement errors). The results are summarized in Table S5. The same as Table 3, we include the variable selection methods Lasso, Lasso Order, RF, GDS, Corrected Lasso, and GMUS. We present the results where the missing probabilities depend on $\mathbf{W}$ rather than $\mathbf{X}$ here. We consider two levels of Scales for the measurement errors. For imputation methods, we consider the default, cart, and rf. We also compare the performance based on whether to impute $\mathbf{Y}$. When the scale of measurement errors is small (Scale = 0.1), all methods control the FDR under the nominal values of 0.2. With bigger scale measurement errors (Scale = 0.6), Lasso Order and RF methods also fail to control the FDR. Corrected Lasso consistently has the lowest FDR, followed by GMUS across all the designed settings. Lasso and GDS also control FDR across all the settings but the FDR are larger than Corrected Lasso and GMUS. In terms of power, the GDS and Lasso methods have the best performance, followed by GMUS and Lasso Order. Corrected Lasso does not have good power. The results for general simultaneous knockoff methods are consistent with the single dataset settings.

Appendix A.4: Additional Simulation from Empirical Data Distributions based on the LC-MS platform (RelQuant).

Setting: We keep the same sample size $n = 1331$ and number of features $p = 148$ as the real data in LC-MS platform (RelQuant).

First, we sample the feature matrix $\mathbf{W}$ by approximating the empirical distribution from real data using the following steps: (1) Sample $\mathbf{W}_e$ from the empirical distribution of $\mathbf{W}$ (i.e., observed transformed metabolites' levels) and sample $\mathbf{E}_e$ from the estimated distribution of measurement error independently. For each column, we sample $W_e(j)$ with the replacement for a sample size $n_{sample} = 10000$ to get $\mathbf{W}_e$. We sample $\mathbf{E}_e \sim \mathcal{N}(\mathbf{0}, \mathbf{\Sigma}_e)$ with the same sample size $n_{sample} = 10000$ where $\mathbf{\Sigma}_e$ is the measure error from the real data. Then we compute $\mathbf{X}_e = \mathbf{W}_e - \mathbf{E}_e$ to obtain the distribution of $\mathbf{X}$ approximately. We denote F_j as the estimated marginal distribution for the $j-$th column of $\mathbf{X}_e$. (2) We sample n individual $\mathbf{Z}_i$ from $\sim \mathcal{N}(\mathbf{0}, \mathbf{\Sigma}_Z)$. Here we let $\mathbf{\Sigma}_Z = AR(\sigma_Z, \rho_Z, p)$, where $AR(\sigma, \rho, p)$ denotes a $p \times p$ matrix with (i,j)-th element equals to $\sigma^2\rho^{|i-j|}$. We use σ^2 to control the magnitude of the variation, and ρ to control the correlation between the predictors. Specifically, we set $\sigma_Z = 1$, $\rho_Z = 0.4$. (3) For each i,j, compute $X_{ij} = F_j^{-1}(\Phi(Z_{ij}))$. (4) We sample $\boldsymbol{\epsilon}_w \sim \mathcal{N}(\mathbf{0}, \sigma_\epsilon^2\mathbf{\Sigma}_e)$, where $\sigma_\epsilon^2 = 0.1$ or 1 controls the scale of measurement errors. (5) We get $\mathbf{W} = \mathbf{X} + \boldsymbol{\epsilon}_w$.

Second, we sample outcome Y_i from a logistic regression model

$$\log \frac{\mathbb{P}(Y_i = 1|\mathbf{X}_i)}{\mathbb{P}(Y_i = 0|\mathbf{X}_i)} = \beta_0 + A_{\boldsymbol{\beta}}\mathbf{X}_i^\top \boldsymbol{\beta} \text{ for } i \in [n].$$

where $\boldsymbol{\beta} = (\mathbf{1}_3 \otimes (3, 1.5, 0, 0, 2, 0, 0), 1, \mathbf{0}_{21}) \odot \epsilon$ is a sparse vector with sparsity level $s = 10$, $A_{\boldsymbol{\beta}} = 1$ controls the magnitude of the effect while ϵ is a vector of independent Rademacher variables, and β_0 controls the prevalence of outcome and is set as $\beta_0 = -1$.

Third, we sample missing data indicators Δ_{ij} under the missing at random (MAR) mechanism. We first randomly choose subsets $S_{mis0} \subset [p] \cap \mathcal{H}$ and $S_{mis1} \subset [p] \cap \mathcal{H}^c$ so that $|S_{mis0}| = \pi_{mis} \cdot (p - s)$ and $|S_{mis1}| = \pi_{mis} \cdot s$ where π_{mis} controls the proportion of variables that will contain missing values and is set at 0.1 to approximate the proportion of variables with more than 5% missing in our real data set. Then for each $j \in S_{mis} = S_{mis0} \cup S_{mis1}$, we sample Δ_{ij} independently from a sequence of logistic regression models

$$\log \frac{\mathbb{P}(\Delta_{ij} = 1|\mathbf{W}_{i,-j})}{\mathbb{P}(\Delta_{ij} = 0|\mathbf{W}_{i,-j})} = \eta_{0j} + \mathbf{W}_{i,-j}^\top \boldsymbol{\eta}_j,$$

where the intercept η_{0j} is used to control the average proportion of missing at 15% and elements of $\boldsymbol{\eta}_j$ equal 1. For $j \notin S_{mis}$, we let $\Delta_{ij} = 1$. Additional simulation when the missing depend on $\mathbf{X}$ rather than $\mathbf{W}$ are also considered where we will then sample Δ_{ij} for each $j \in S_{mis}$ by

$$\log \frac{\mathbb{P}(\Delta_{ij} = 1|\mathbf{X}_{i,-j})}{\mathbb{P}(\Delta_{ij} = 0|\mathbf{X}_{i,-j})} = \eta_{0j} + \mathbf{X}_{i,-j}^\top \boldsymbol{\eta}_j,$$

where the intercept η_{0j} is used to control the average proportion of missing among those variables with missing value at $p_{mis} = 15\%$ and elements of $\boldsymbol{\eta}_j$ equal 1.

Results: Under this setting, we compare the performance of the following methods: Lasso, Lasso Order, RF, GDS, GMUS, and Corrected Lasso with respect to FDR control and power calculated from 100 replicates when using multiple imputations from chained equations with 5 imputed datasets each.

Considering that the Missing At Random (MAR) assumption, predicated on error-prone variables ($\mathbf{W}$), may be overly stringent for certain applications, we conducted a simulation study to evaluate the sensitivity of our results to this assumption. Specifically, we investigated scenarios where the missing probability is contingent on the error-free variables ($\mathbf{X}$), as opposed to $\mathbf{W}$. The outcomes of this investigation are succinctly summarized in Table S6. The results are consistent

with what we found when missingness depended on error-prone variables ($\mathbf{W}$). This analysis is crucial for understanding the robustness of our findings under different assumptions regarding the nature of the missing data mechanism.

Appendix B: Additional data analysis details

Appendix B.1: Matching Method

Cases and controls for this analysis were selected from the entire Women's Health Initiative (WHI) Bone Mineral Density (BMD) Subcohort (n=11,020). The BMD was comprised of women in both the Clinical Trial (CT) and Observational Study (OS), who were enrolled at three specified WHI clinical centers (Birmingham, Pittsburgh, and Tucson/Phoenix), had dual X-ray absorptiometry at baseline and follow-up time points, and provided spot urine specimens, as well as fasting blood samples. For the CT samples, they include both dietary modification trials (DM) and hormone therapy trials (HT). Here the eligible sample was restricted to women who had sufficient WHI serum (300 μl) and urine (550 μl) samples from the same time point, before and closest to the case diagnosis date and were required to have no missing covariate data (n=10,451). The cases were defined as the earliest incident invasive breast cancer (BC) or colorectal cancer (CRC) so that the biospecimen collection would be reasonably proximate. Each of the 758 case women was matched 1-to-1 to a control woman, disease-free at the case occurrence follow-up time, based on age (within 2 years; Table I), WHI enrollment date (within 2 months to control for follow-up duration), and race/ethnicity. Participants could only be a control for one case, and a case could not be a control for another case. The matching algorithm was applied to select the closest match based on criteria to minimize an overall distance measure (Bergstralh and Kosanke, 1995). Each matching factor was given the same weight. Controls were excluded for the following reasons: a) history of BC or CRC reported at baseline (n=382); b) no follow-up (n=32); c) missing any covariate data (n=3513 breast, n=2905 colorectal). The number of eligible controls was n=6477 BC controls, n=7056 CRC controls.

Because these two control groups overlap; the 181 CRC cases were matched first, matched controls were removed from the eligible pool, then BC cases were matched. 54% of the selected sample were in the OS, 34% in the DM, and 12% in the HT-not DM.

Appendix B.2: Data preprocessing

Data are analyzed separately for each metabolomics platform. For metabolic variables, we removed those with more than 20% missing values. We take log transformation to all lab-measured variables to be consistent with other analyses in the Nutrition and Physical Activity Assessment Study Feeding Study (NPAAS-FS) Zheng et al. (2021). Outliers were truncated to Q1-3*IQR or Q3+3*IQR where Q1 and Q3 are the first and the third quartiles and IQR is the interquartile range. To remove the batch and run order effect for LC-MS and GC-MS data, normalization was performed using local polynomial regression fitting over run order within each batch.

Appendix B.3: Calculation of variance-covariance matrix for the measurement errors

To calculate the variance-covariance matrix for the measurement errors, we utilize the QC samples. Specifically, we use pooled NPAAS-FS first void urine QC samples for the GC-MS platform

and NMR platform and we use pooled NPAAS and NPAAS-FS serum QC samples for the LC-MS platform and the lipidomic platform. Log transformation is also performed on all the lab-measured variables to be consistent with non-QC samples. We also remove variables with more than 20% missing values in non-QC samples and all missings in QC samples. The same normalization method is also performed for QC samples. For GC-MS and NMR, we use the sample variance and covariance matrix of the QC samples to estimate the variance-covariance matrix for the measurement errors. For lipidomic and NMR platforms, QC samples are collected twice for each batch. To fully remove the batch effect, we use half of the sample variance-covariance matrix of the difference within each batch to estimate the variance-covariance matrix for the measurement error. If the estimated variance-covariance matrix as above is not positive definite, we add a data-adaptive value (i.e., the first eigenvalue less than a threshold 10^{-4}) to the corresponding correlation matrix and re-calculate the covariance matrix based on the new correlation matrix and original standard deviations. The summary information of missing and measurement error for each platform (after removing those with $> 20\%$ missing) is shown in Table S7.

Appendix C: Additional data analysis results

In this section, we first provide the detailed list of metabolites selected for at least 10% of times from the stability selection for each method and each cancer outcome separately. Then we provide the detailed list of metabolites selected for at least 10% of times from the stability selection for associated with both breast and colorectal cancer using preferred methods (Lasso, Lasso order, and GMUS). Finally, we provide the selected for at least 50% of times from the stability selection for the preferred method and each cancer outcome separately when using only matched controls specific to that cancer.

Data analysis results using Lasso

Metabolites that are selected using Lasso among $\geqslant$10% of replications associated with BC and CRC are listed in Tables S8 and S9.

Data analysis results using Lasso Order

Metabolites that are selected using Lasso Order among $\geqslant$10% of replications associated with BC and CRC are listed in Tables S10 and S11.

Data analysis results using Random Forest

Metabolites that are selected using RF among $\geqslant$10% of replications associated with BC and CRC are listed in Tables S12 and S13.

Data analysis results using GDS

Metabolites that are selected using GDS among $\geqslant$10% of replications associated with BC and CRC are listed in Tables S14 and S15.

Data analysis results using GMUS

Metabolites that are selected using GMUS among $\geqslant$10% of replications associated with BC and CRC are listed in Tables S16 and S17.

Data analysis results using Corrected Lasso

Metabolites that are selected using Corrected Lasso among ⩾10% of replications associated with BC and CRC are listed in Tables S18 and S19.

Metabolites selected for more than 10% of times that are associated with both BC and CRC.

Tables S20, S21 and S22 provide the variables selected from at least 10% of the replications and the corresponding percentage time of selection for mutual risk factor analysis.

Sensitivity analysis using cancer-specific matched controls

Metabolites that are robustly (⩾50% times selected) associated with BC, CRC, and mutual risks and the direction of their marginal association to the BC/CRC/mutual risks using the corresponding cancer specific matched controls are listed in Tables S23, S24 and S25.

References

Bergstralh EJ, Kosanke JL (1995). Computerized matching of cases to controls. In: *Mayo Clinic.*

Zheng C, Gowda G, Raftery D, Neuhouser M, Tinker L, Prentice R, et al. (2021). Evaluation of potential metabolomic-based biomarkers of protein, carbohydrate and fat intakes using a controlled feeding study. *European Journal of Nutrition*, 60(8): 4207–4218.

Table S1: Simulation results for Prediction Performance of Random Forest for Setting 1 (missing data only) varying n, p and p_{mis}; with different imputation methods (Imp M) and choice of whether to include Y in the imputation (Imp Y).

					Performance				
n	p	p_{mis}	Imp M	Imp Y	Accuracy	Precision	Recall	F1	AUC
400	60	0.05	cart	yes	0.76	0.80	0.64	0.70	0.85
400	60	0.05	cart	no	0.76	0.80	0.64	0.70	0.85
400	60	0.05	default	yes	0.76	0.80	0.64	0.70	0.85
400	60	0.05	default	no	0.75	0.80	0.63	0.70	0.85
400	60	0.05	rf	yes	0.75	0.80	0.64	0.70	0.85
400	60	0.05	rf	no	0.76	0.80	0.64	0.70	0.85
400	60	0.15	cart	yes	0.75	0.79	0.63	0.69	0.85
400	60	0.15	cart	no	0.75	0.79	0.64	0.69	0.85
400	60	0.15	default	yes	0.76	0.80	0.64	0.70	0.85
400	60	0.15	default	no	0.75	0.79	0.64	0.70	0.85
400	60	0.15	rf	yes	0.75	0.80	0.64	0.70	0.85
400	60	0.15	rf	no	0.75	0.79	0.63	0.70	0.85
1000	60	0.05	cart	yes	0.79	0.82	0.70	0.75	0.89
1000	60	0.05	cart	no	0.79	0.82	0.70	0.75	0.88
1000	60	0.05	default	yes	0.79	0.82	0.70	0.75	0.89
1000	60	0.05	default	no	0.79	0.82	0.70	0.75	0.88
1000	60	0.05	rf	yes	0.79	0.82	0.70	0.75	0.88
1000	60	0.05	rf	no	0.79	0.82	0.70	0.75	0.88
1000	60	0.15	cart	yes	0.79	0.82	0.70	0.75	0.88
1000	60	0.15	cart	no	0.79	0.81	0.70	0.75	0.88
1000	60	0.15	default	yes	0.80	0.82	0.70	0.76	0.89
1000	60	0.15	default	no	0.79	0.81	0.70	0.75	0.88
1000	60	0.15	rf	yes	0.79	0.82	0.70	0.75	0.88
1000	60	0.15	rf	no	0.79	0.81	0.70	0.75	0.88
1000	120	0.05	cart	yes	0.76	0.81	0.65	0.72	0.86
1000	120	0.05	cart	no	0.76	0.81	0.65	0.72	0.86
1000	120	0.05	default	yes	0.76	0.81	0.65	0.72	0.86
1000	120	0.05	default	no	0.76	0.81	0.65	0.72	0.86
1000	120	0.05	rf	yes	0.76	0.81	0.65	0.72	0.86
1000	120	0.05	rf	no	0.76	0.81	0.65	0.71	0.86
1000	120	0.15	cart	yes	0.76	0.81	0.65	0.72	0.85
1000	120	0.15	cart	no	0.76	0.80	0.65	0.71	0.85
1000	120	0.15	default	yes	0.76	0.81	0.65	0.72	0.86
1000	120	0.15	default	no	0.76	0.80	0.65	0.72	0.85
1000	120	0.15	rf	yes	0.76	0.81	0.65	0.71	0.85
1000	120	0.15	rf	no	0.76	0.80	0.65	0.71	0.85

Table S2: Simulation results (FDP and Power) for Setting 3 (data with both measurement errors and missing data) for $n = 1000$, $\sigma_\epsilon^2 = 0.6$, $p_{mis} = 0.15$, and $A_{\boldsymbol{\beta}} = 1$, varying p, Imp M and Imp Y when the missing probability depends on the error-prone variables $\mathbf{W}$.

FDP								
p	Imp M	Imp Y	Lasso	Lasso Order	RF	GDS	Corrected Lasso	GMUS
60	default	yes	0.20	0.25	0.25	0.20	0.19	0.17
60	default	no	0.18	0.26	0.27	0.20	0.21	0.17
60	cart	yes	0.20	0.26	0.26	0.20	0.19	0.18
60	cart	no	0.19	0.28	0.26	0.21	0.18	0.19
60	rf	yes	0.20	0.27	0.28	0.21	0.20	0.16
60	rf	no	0.19	0.28	0.26	0.20	0.18	0.18
210	default	yes	0.19	0.20	0.19	0.17	0.00	0.19
210	default	no	0.17	0.21	0.20	0.17	0.01	0.16
210	cart	yes	0.18	0.21	0.18	0.18	0.01	0.17
210	cart	no	0.19	0.22	0.19	0.19	0.00	0.17
210	rf	yes	0.19	0.22	0.21	0.17	0.01	0.17
210	rf	no	0.19	0.22	0.20	0.18	0.00	0.17
Power								
p	Imp M	Imp Y	Lasso	Lasso Order	RF	GDS	Corrected Lasso	GMUS
60	default	yes	0.94	0.86	0.78	0.93	0.61	0.93
60	default	no	0.93	0.85	0.77	0.93	0.61	0.91
60	cart	yes	0.94	0.86	0.78	0.93	0.57	0.92
60	cart	no	0.93	0.86	0.77	0.93	0.56	0.92
60	rf	yes	0.93	0.86	0.78	0.92	0.60	0.92
60	rf	no	0.93	0.86	0.77	0.93	0.58	0.91
210	default	yes	0.65	0.48	0.35	0.57	0.01	0.60
210	default	no	0.63	0.48	0.35	0.57	0.02	0.56
210	cart	yes	0.64	0.49	0.36	0.61	0.02	0.59
210	cart	no	0.64	0.52	0.37	0.61	0.01	0.59
210	rf	yes	0.65	0.50	0.39	0.58	0.02	0.60
210	rf	no	0.64	0.51	0.38	0.58	0.01	0.60

Table S3: Simulation results (FDP and Power) of different methods for Setting 3 (data with both measurement errors and missing data) for $n = 1000$, $p = 60$, $p_{mis} = 0.15$, and $A_{\boldsymbol{\beta}} = 1$, varying σ_ϵ^2, Imp M and Imp Y when the missing probability depends on the error-prone variables when the missing probability depends on the error-free variables $\mathbf{X}$.

FDP								
σ_ϵ^2	Imp M	Imp Y·	Lasso	Lasso Order	RF	GDS	Corrected Lasso	GMUS
0.1	default	yes	0.34	0.23	0.12	0.36	0.04	0.31
0.1	default	no	0.32	0.22	0.14	0.33	0.03	0.28
0.1	cart	yes	0.32	0.24	0.12	0.33	0.04	0.28
0.1	cart	no	0.30	0.23	0.15	0.32	0.03	0.26
0.1	rf	yes	0.34	0.24	0.15	0.34	0.04	0.29
0.1	rf	no	0.32	0.25	0.15	0.32	0.02	0.29
0.6	default	yes	0.57	0.26	0.10	0.54	0.07	0.52
0.6	default	no	0.56	0.27	0.13	0.50	0.07	0.49
0.6	cart	yes	0.56	0.32	0.13	0.54	0.08	0.50
0.6	cart	no	0.56	0.30	0.12	0.52	0.08	0.50
0.6	rf	yes	0.56	0.32	0.13	0.54	0.07	0.49
0.6	rf	no	0.56	0.30	0.13	0.54	0.07	0.50
Power								
σ_ϵ^2	Imp M	Imp Y	Lasso	Lasso Order	RF	GDS	Corrected Lasso	GMUS
0.1	default	yes	1.00	0.93	0.76	1.00	0.41	1.00
0.1	default	no	1.00	0.91	0.77	1.00	0.36	1.00
0.1	cart	yes	1.00	0.92	0.78	1.00	0.37	1.00
0.1	cart	no	1.00	0.91	0.78	1.00	0.37	1.00
0.1	rf	yes	1.00	0.93	0.79	1.00	0.37	1.00
0.1	rf	no	1.00	0.93	0.78	1.00	0.33	1.00
0.6	default	yes	0.93	0.70	0.52	0.93	0.32	0.92
0.6	default	no	0.92	0.73	0.56	0.92	0.29	0.92
0.6	cart	yes	0.94	0.73	0.59	0.94	0.31	0.93
0.6	cart	no	0.94	0.74	0.56	0.94	0.29	0.93
0.6	rf	yes	0.94	0.77	0.60	0.94	0.30	0.93
0.6	rf	no	0.94	0.75	0.58	0.94	0.29	0.93

Table S4: Oracle results (FDP and Power) for Setting 3 (data with both measurement errors and missing data) for $n = 1000$, $p = 60$, $p_{mis} = 0.15$, and $A_{\boldsymbol{\beta}} = 1$, varying σ_ϵ^2, and the missing probability depends on the error-prone variables $\mathbf{W}$ or the error-free variables $\mathbf{X}$.

σ_ϵ^2	Type	FDP	Power
0.1	**W**	0.33	0.95
0.6	**W**	0.34	0.87
0.1	**X**	0.34	0.97
0.6	**X**	0.35	0.95

Table S5: Simulation results (FDP and Power) for Simultaneous Knockoff Methods for Two datasets with both measurement errors and missing data) for $n = 1000$, $p = 60$, $p_{mis} = 0.15$, and $A_{\boldsymbol{\beta}} = 1$, varying σ_ϵ^2, Imp M and Imp Y when the missing probability depends on the error-prone variables $\mathbf{W}$.

FDP								
σ_ϵ^2	Imp M	Imp Y	Lasso	Lasso Order	RF	GDS	Corrected Lasso	GMUS
0.1	default	yes	0.09	0.18	0.18	0.11	0.00	0.05
0.1	default	no	0.08	0.19	0.17	0.10	0.00	0.05
0.1	cart	yes	0.08	0.18	0.17	0.11	0.00	0.05
0.1	cart	no	0.08	0.19	0.18	0.10	0.00	0.04
0.1	rf	yes	0.10	0.19	0.18	0.12	0.00	0.05
0.1	rf	no	0.10	0.18	0.18	0.12	0.00	0.05
0.6	default	yes	0.11	0.21	0.21	0.12	0.07	0.07
0.6	default	no	0.11	0.22	0.23	0.12	0.08	0.07
0.6	cart	yes	0.11	0.22	0.22	0.12	0.09	0.09
0.6	cart	no	0.10	0.21	0.22	0.14	0.07	0.08
0.6	rf	yes	0.11	0.23	0.21	0.13	0.08	0.09
0.6	rf	no	0.12	0.24	0.23	0.14	0.08	0.08
Power								
σ_ϵ^2	Imp M	Imp Y	Lasso	Lasso Order	RF	GDS	Corrected Lasso	GMUS
0.1	default	yes	1.00	0.96	0.85	1.00	0.22	0.98
0.1	default	no	1.00	0.96	0.85	1.00	0.19	0.96
0.1	cart	yes	1.00	0.95	0.85	1.00	0.19	0.96
0.1	cart	no	1.00	0.96	0.84	1.00	0.20	0.95
0.1	rf	yes	1.00	0.95	0.85	1.00	0.22	0.96
0.1	rf	no	1.00	0.95	0.86	1.00	0.21	0.96
0.6	default	yes	0.94	0.87	0.82	0.94	0.42	0.89
0.6	default	no	0.93	0.87	0.81	0.93	0.42	0.89
0.6	cart	yes	0.93	0.87	0.81	0.93	0.42	0.89
0.6	cart	no	0.93	0.87	0.82	0.93	0.39	0.89
0.6	rf	yes	0.93	0.87	0.82	0.93	0.41	0.89
0.6	rf	no	0.93	0.87	0.82	0.93	0.42	0.89

Table S6: Simulation results (FDP and Power) for Empirical Data Distribution from LC-MS platform (RelQuant) for $n = 1331$, $p = 148$, $p_{mis} = 0.15$, and $A_{\boldsymbol{\beta}} = 1$, varying σ_ϵ^2, Imp M and Imp Y when the missing probability depends on the error-prone variables $\mathbf{X}$.

FDP								
σ_ϵ^2	Imp M	Imp Y	Lasso	Lasso Order	RF	GDS	Corrected Lasso	GMUS
0.1	default	yes	0.18	0.18	0.15	0.19	0.03	0.20
0.1	default	no	0.18	0.17	0.14	0.19	0.04	0.19
0.1	cart	yes	0.17	0.16	0.15	0.19	0.05	0.19
0.1	cart	no	0.17	0.17	0.14	0.20	0.05	0.18
0.1	rf	yes	0.18	0.18	0.14	0.20	0.05	0.19
0.1	rf	no	0.18	0.18	0.15	0.19	0.02	0.18
0.5	default	yes	0.20	0.19	0.14	0.23	0.05	0.19
0.5	default	no	0.20	0.19	0.14	0.21	0.04	0.19
0.5	cart	yes	0.19	0.18	0.15	0.20	0.04	0.20
0.5	cart	no	0.19	0.19	0.15	0.20	0.05	0.19
0.5	rf	yes	0.20	0.19	0.15	0.21	0.04	0.20
0.5	rf	no	0.19	0.20	0.15	0.19	0.05	0.20
Power								
σ_ϵ^2	Imp M	Imp Y	Lasso	Lasso Order	RF	GDS	Corrected Lasso	GMUS
0.1	default	yes	1.00	0.95	0.72	1.00	0.11	1.00
0.1	default	no	1.00	0.93	0.70	1.00	0.10	1.00
0.1	cart	yes	1.00	0.92	0.71	1.00	0.11	1.00
0.1	cart	no	1.00	0.92	0.71	1.00	0.12	1.00
0.1	rf	yes	1.00	0.93	0.71	1.00	0.12	1.00
0.1	rf	no	1.00	0.94	0.72	1.00	0.10	1.00
0.5	default	yes	1.00	0.94	0.72	1.00	0.29	1.00
0.5	default	no	1.00	0.93	0.70	1.00	0.28	1.00
0.5	cart	yes	1.00	0.93	0.72	1.00	0.28	1.00
0.5	cart	no	1.00	0.93	0.71	1.00	0.29	1.00
0.5	rf	yes	1.00	0.93	0.72	1.00	0.28	1.00
0.5	rf	no	1.00	0.93	0.71	1.00	0.28	1.00

Table S7: Summary information of missing and measurement errors for each platform

Platform	Number of variables having missing N (%)	The proportion (%) of missing Mean (SD)	$SNR^{\dagger}$ Median, [Q1, Q3] (Min−Max)
NMR	10 (16.95)	2.36 (2.67)	100.7, [58.2, 169.1] (8.0−1873.3)
GC-MS	58 (86.57)	6.25 (6.05)	34.0, [14.8, 92.1] (0.1−1397328.9)
LC-MS (AbsQuant)	23 (76.67)	0.07 (0)	37.3, [13.8, 79.0] (1.0−1786.6)
LC-MS (RelQuant)	91 (61.69)	1.36 (3.6)	92.4, [31.3, 251.8] (0.2−44362.3)
Lipidyzer (composition)	413 (60.82)	2.14 (3.61)	37.5, [14.8, 100.0] (0.9−2133.4)
Lipidyzer (concentration)	413 (60.82)	2.14 (3.61)	76.8, [28.3, 228.5] (1.9−2695.9)

SNR is signal noise ratio defined as $Var(X)/Var(\epsilon_W)$.

Table S8: Metabolites that are selected using **Lasso** among ⩾10% of replications associated with BC risks and the direction of their marginal association to the BC risks.

Platform	Half Min Imputation	Multiple Imputation
NMR		
GC-MS	Alpha−ketoglutarate (76%)(-)	
LC-MS (AbsQuant)	Choline (13%)(+)<br>3HBA (13%)(+)	Choline (13%)(+)
LC-MS (RelQuant)		
Lipidyzer (composition)	DAG 14:1/18:1 (40%)(+)<br>TAG 47:0(FA15:0)) (39%)(-)<br>TAG 48:3 (FA18:1) (24%)(-)	TAG 48:5(FA18:3) (80%)(-)<br>DAG 14:1/18:1 (78%)(+)<br>TAG 48:0(FA16:0)(20%)(+)
Lipidyzer (concentration)	DAG 14:1/18:1 (97%)(+)<br>TAG 48:0(FA16:0) (49%)(+)<br>TAG 56:9(FA20:4) (17%)(-)<br>PE 18:1/20:3 (14%)(-)	DAG 14:1/18:1 (97%)(+)<br>TAG48:5(FA18.3) (64%)(-)<br>PE 18:2/20:4 (11%)(-)

Table S9: Metabolites that are selected using **Lasso** among ⩾10% of replications associated with CRC risks and the direction of their marginal association to the CRC risks.

Platform	Half Min Imputation	Multiple Imputation
NMR	N−methylnicotinic acid (43%)(-)<br>Taurine (22%)(+)	Taurine (16%)(+)
GC-MS	2,3−Dihydroxybutanoic acid (46%)(+)	2,3−Dihydroxybutanoic acid (93%)(+)
LC-MS (AbsQuant)	Glucose (84%)(+)<br>Cystine (52%)(-)<br>Serine (44%)(+)<br>Urate (42%)(+)<br>Choline (32%)(+)<br>Glycine (18%)(+)<br>Proline (11%)(+)	Glucose (62%)(+)<br>Serine (43%)(+)<br>Urate (14%)(+)<br>Choline (10%)(+)
LC-MS (RelQuant)	Glycerate (69%)(+)<br>Adenosine (66%)(-)<br>Adipic Acid (14%)(+)	Adenosine (69%)(-)<br>Glycerate (67%)(+)
Lipidyzer (composition)	TAG 48:5(FA18:2) (59%)(+)<br>TAG 47:2(FA14:0) (36%)(-)<br>TAG 52:8(FA16:1) (32%)(-)<br>TAG 54:0(FA16:0) (25%)(-)<br>TAG 46:4(FA18:2) (17%)(+)	
Lipidyzer (concentration)		

Table S10: Metabolites that are selected using **Lasso Order** among ⩾10% of replications associated with BC risks and the direction of their marginal association to the BC risks.

Platform	Half Min Imputation	Multiple Imputation
NMR	N−methylnicotinic acid (59%)(+)	N−methylnicotinic acid (63%)(+)
GC-MS	Alpha−ketoglutarate (54%)(-)	2,3−Dihydroxybutanoic acid (42%)(-)
LC-MS (AbsQuant)	3HBA (67%)(+)<br>Cystine (66%)(-)	Cystine (34%)(-)<br>3HBA (19%)(+)
LC-MS (RelQuant)	Malate (45%) (-)	Malate (47%)(-)
Lipidyzer (composition)	TAG 47:0(FA15:0) (68%)(-)	TAG 48:5(FA18:3) (80%)(-)<br>DAG 14:1/18:1 (76%)(+)<br>TAG 58:10(FA20:5) (37%)(-)<br>TAG 56:9(FA20:4) (21%)(-)<br>TAG 46:3(FA16:1) (16%)(-)
Lipidyzer (concentration)	TAG 44:1(FA12:0) (24%)(-)	

Table S11: Metabolites that are selected using **Lasso Order** among ⩾10% of replications associated with CRC risks and the direction of their marginal association to the CRC risks.

Platform	Half Min Imputation	Multiple Imputation
NMR	N−methylnicotinic acid (76%)(-)	N−methylnicotinic acid (54%)(-)
GC-MS	2,3−Dihydroxybutanoic acid (46%)(+)	2,3−Dihydroxybutanoic acid (63%)(+)
LC-MS (AbsQuant)	Cystine (100%)(-)<br>3HBA (75%)(+)<br>Glutamic acid (18%)(+)<br>Glucose (16%)(+)<br>Glycine (12%)(+)<br>Choline (12%)(+)<br>Urate (12%)(+)	Cystine (94%)(-)<br>3HBA (31%)(+)
LC-MS (RelQuant)	Malate (57%)(+)	Malate (49%)(+)
Lipidyzer (composition)	TAG 48:5(FA18:2) (60%)(+)<br>TAG 54:0(FA16:0) (40%)(-)<br>TAG 52:8(FA16:1) (34%)(-)<br>TAG 47:2(FA14:0) (20%)(-)<br>TAG 46:4(FA18:2) (13%)(+)	TAG 48:4(FA14:0) (21%)(-)
Lipidyzer (concentration)	TAG 47:2(FA14:0) (61%)(-)	

Table S12: Metabolites that are selected using **Random Forest** among ⩾10% of replications associated with BC risks and the direction of their marginal association to the BC risks.

Platform	Half Min Imputation	Multiple Imputation
NMR	Uracil (72%)(-)	Uracil (35%)(-)
GC-MS		
LC-MS (AbsQuant)	Choline (38%)(+)<br>Citrulline (32%)(-)<br>Cystine (18%)(-)<br>3HBA (11%)(+)	Choline (12%)(+)
LC-MS (RelQuant)		Glycochenodeoxycholate (33%)(+)
Lipidyzer (composition)	DAG 14:1/18:1 (44%)(+)<br>PE 18:0/20:2 (25%)(+)	DAG 14:1/18:1 (23%)(+)
Lipidyzer (concentration)	DAG 14:1/18:1 (67%)(+)<br>PEP 18:1/22:5 (16%)(-)	DAG 14:1/18:1 (82%)(+)<br>PE 18:2/20:4 (13%)(-)<br>PEO 18:0/18:1 (11%)(-)<br>PC 18:0/20:0 (10%)(-)

Table S13: Metabolites that are selected using **Random Forest** among ⩾10% of replications associated with CRC risks and the direction of their marginal association to the CRC risks.

Platform	Half Min Imputation	Multiple Imputation
NMR		
GC-MS		
LC-MS (AbsQuant)	Glucose (73%)(+)<br>Cystine (55%)(-)<br>Pentothenate (54%)(+)<br>Histidine (43%)(-)<br>Threonine (19%)(-)<br>Serine (11%)(+)	Glucose (73%)(+)<br>Histidine (44%)(-)<br>Cystine (44%)(-)<br>Pentothenate (35%)(+)<br>Threonine (20%)(-)
LC-MS (RelQuant)		Adenosine (24%)(-)
Lipidyzer (composition)	LCER 16:0 (14%)(-)<br>PC 18:0/18:0 (13%)(+)<br>TAG 50:5(FA18:1) (10%)(-)	PC 18:0/18:0 (28%)(+)<br>LCER 16:0 (23%)(-)
Lipidyzer (concentration)	PC 18:1/18:3 (21%)(-)<br>LPE 18:0 (14%)(+)<br>PC 18:2/18:3 (13%)(-)	PEO 16:0/18:2 (14%)(-)<br>LPE 20:4 (12%)(+)<br>PC 18:1/18:3 (11%)(-)

Table S14: Metabolites that are selected using **GDS** among ⩾10% of replications associated with BC risks and the direction of their marginal association to the BC risks.

Platform	Half Min Imputation	Multiple Imputation
NMR	Uracil (21%)(-)<br>Formate (19%)(+)	
GC-MS		
LC-MS (AbsQuant)	Choline (61%)(+)	Choline (27%)(+)
LC-MS (RelQuant)		
Lipidyzer (composition)	TAG 52:2(FA18:2) (68%)(+)<br>PE 18:1/20:3 (36%)(-)<br>TAG 50:4(FA18:1) (20%)(-)	PC 16:0/18:2 (52%)(+)<br>TAG 52:2(FA18:2) (34%)(+)<br>FFA 20:2 (13%)(+)
Lipidyzer (concentration)	DAG 14:1/18:1 (64%)(+)<br>PC 18:1/22:5 (40%)(-)<br>SM 20:0 (25%)(+)<br>FFA 20:2 (12%)(+)	DAG 14:1/18:1 (86%)(+)<br>PE 18:2/20:4 (44%)(-)<br>SM 20:0 (42%)(+)<br>PC 18:1/22:5 (34%)(-)

Table S15: Metabolites that are selected using **GDS** among ⩾10% of replications associated with CRC risks and the direction of their marginal association to the CRC risks.

Platform	Half Min Imputation	Multiple Imputation
NMR	Taurine (51%)(+)<br>Histidine (24%)(-)	Taurine (14%)(+)
GC-MS		
LC-MS (AbsQuant)	Serine (90%)(+)<br>Histidine (89%)(-)<br>Choline (60%)(+)<br>Glucose (55%)(+)<br>Urate (40%)(+)<br>Glutamic acid (17%)(+)	Histidine (90%)(-)<br>Serine (74%)(+)<br>Glucose (33%)(+)<br>Choline (25%)(+)<br>Urate (15%)(+)<br>Glutamic acid (11%)(+)
LC-MS (RelQuant)	Adenosine (76%)(-)<br>Glycerate (19%)(+)	Adenosine (84%)(-)
Lipidyzer (composition)	HCER 24:0 (21%)(-)<br>TAG 54:2(FA18:1) (18%)(+)	
Lipidyzer (concentration)	CER 16:0 (53%)(+)<br>PC 18:2/20.3 (20%)(-)<br>CER 24:1 (18%)(+)	CER 16:0 (70%)(+)<br>PC 18:2/20:3 (11%)(-)

Table S16: Metabolites that are selected using **GMUS** among ⩾10% of replications associated with BC risks and the direction of their marginal association to the BC risks.

Platform	Half Min Imputation	Multiple Imputation
NMR	Uracil (19%)(-)<br>Formate (11%)(+)	
GC-MS		
LC-MS (AbsQuant)	Choline (46%)(+)	Choline (38%)(+)
LC-MS (RelQuant)		
Lipidyzer (composition)	TAG 52:2(FA18:2) (66%)(-)<br>PE 18:1/20:3 (21%)(-)	PC 16:0/18:2 (52.6%)(+)<br>TAG 52:2(FA18:2) (24.2%)(+)
Lipidyzer (concentration)	DAG 14:1/18:1 (63%)(+)<br>PC 18:1/22:5 (42%)(-)<br>TAG 54:8(FA20:4)(13%)(-)<br>FFA 20:2 (11%)(+)<br>SM 20:0 (11%)(+)	DAG 14:1/18:1 (93.3%)(+)<br>PE 18:2/20:4 (37.8%)(-)<br>PC 18:1/22:5 (31.1%)(-)<br>SM 20:0 (13.3%)(+)

Table S17: Metabolites that are selected using **GMUS** among ⩾10% of replications associated with CRC risks and the direction of their marginal association to the CRC risks.

Platform	Half Min Imputation	Multiple Imputation
NMR	Taurine (35%)(+)<br>Histidine (19%)(-)	Taurine (28%)(+)
GC-MS		
LC-MS (AbsQuant)	Choline (42%)(+)<br>Glucose (39%)(+)<br>Serine (35%)(+)<br>Histidine (35%)(-)<br>Cystine (31%)(-)<br>Glutamic acid (25%)(+)<br>Urate (12%)(+)	Histidine (69%)(-)<br>Serine (46%)(+)<br>Glucose (25%)(+)<br>Choline (23%)(+)<br>Glutamic acid (16%)(+)
LC-MS (RelQuant)	Adenosine (42%)(-)<br>Glycerate (28%)(+)	Adenosine (29%)(-)<br>Glycerate (11%)(+)
Lipidyzer (composition)	HCER 24:0 (12%)(-)<br>TAG 54:2(FA18:1) (10%)(+)	
Lipidyzer (concentration)	CER 16:0 (52%)(+)<br>PC 18:2/20:3 (22%)(-)<br>CER 24:1 (15%)(+)	CER 16:0 (66%)(+)

Table S18: Metabolites that are selected using **Corrected Lasso** among ⩾10% of replications associated with BC risks and the direction of their marginal association to the BC risks.

Platform	Half Min Imputation	Multiple Imputation
NMR	N−methylnicotinic acid (95%)(+)	N−methylnicotinic acid (99%)(+)
GC-MS	Alpha−ketoglutarate (55%)(-)<br>2,3−Dihydroxybutanoic acid (35%)(-)<br>Serine (21%)(-)<br>Phenol (15%)(-)	Alpha−ketoglutarate (73%)(-)<br>2,3−Dihydroxybutanoic acid (11%)(-)
LC-MS (AbsQuant)	Aspartic Acid (17%)(+)<br>Glucose (15%)(+)<br>Cystine (11%)(-)	
LC-MS (RelQuant)	Ribose−5−P (43%)(-)<br>Malate (12%)(-)	Ribose−5−P (30%)(-)
Lipidyzer (composition)	DAG 14:1/18:1 (25%)(+)<br>TAG 47:0(FA15:0) (18%)(-)<br>PC 18:1/22:4 (13%)(-)<br>PEP 18:1/22:4 (13%)(+)<br>DAG 16:1/18:1 (12%)(-)<br>DAG 18:0/18:1 (12%)(+)<br>DAG 18:2/20:4 (10%)(-)<br>TAG 44:0(FA16:0) (10%)(-)<br>TAG 44:1(FA12:0) (10%)(-)	
Lipidyzer (concentration)	TAG 44:0(FA16:0) (34%)(-)<br>TAG 44:1(FA12:0) (15%)(-)<br>TAG 44:0(FA14:0) (13%)(-)<br>TAG 46:0(FA16:0) (12%)(-)	TAG 44:0(FA16:0) (32%)(-)<br>TAG 46:1(FA16:1) (10%) (-)

Table S19: Metabolites that are selected using **Corrected Lasso** among ⩾10% of replications associated with CRC risks and the direction of their marginal association to the CRC risks.

Platform	Half Min Imputation	Multiple Imputation
NMR	N−methylnicotinic acid (86%)(-)	Taurine (86%)(+)
GC-MS	2,3−Dihydroxybutanoic acid (47%)(+)<br>Phenol, 2,4−bis(1,1−dimethylethyl)−, phosphite (3:1)(22%)(+)	Pseudo uridine penta−tms (20%)(+)<br>2,3−Dihydroxybutanoic acid (17%)(+)<br>Alpha−ketoglutarate (15%)(+)<br>4,5−dihydroxy−1,2−dithiane (14%)(+)
LC-MS (AbsQuant)	Methionine (46%)(-)<br>Glucose (29%)(+)	Methionine (59%)(-)<br>Glucose (59%)(+)<br>iso−Leucine (23%)(+)<br>Leucine (15%)(+)
LC-MS (RelQuant)	Malate (71%)(+)	Malate (87%)(+)
Lipidyzer (composition)	TAG 44:0(FA16:0) (15%)(+)<br>TAG 44:0(FA14:0) (14%)(-)<br>TAG 46:0(FA14:0) (11%)(-)<br>TAG 46:0(FA16:0) (11%)(-)	
Lipidyzer (concentration)	TAG 44:0(FA16:0) (43%)(-)<br>TAG 44:0(FA14:0) (21%)(-)<br>TAG 44:1(FA14:0) (11%)(-)	

Table S20: Metabolites that are selected using **Lasso** among ⩾10% of replications associated with both BC and CRC risks and the direction of their marginal association to these two cancer risks.

Platform	Half Min Imputation	Multiple Imputation
NMR		
GC-MS		
LC-MS (AbsQuant)	Cystine (44%)(B:-)(C:-)<br>Choline (39%)(B:+)(C:+)<br>3HBA (31%)(B:+)(C:+)<br>Glutamic acid (16%)(B:+)(C:+)	Choline (17%)(B:+)(C:+)
LC-MS (RelQuant)	Malate (11%)(B:-)(C:+)	
Lipidyzer (composition)		
Lipidyzer (concentration)		

Table S21: Metabolites that are selected using **Lasso Order** among ⩾10% of replications associated with both BC and CRC risks and the direction of their marginal association to these two cancer risks.

Platform	Half Min Imputation	Multiple Imputation
NMR	N−methylnicotinic acid (48%)(B:+)(C:-)	N−methylnicotinic acid (57%)(B:+)(C:-)
GC-MS	2,3−Dihydroxybutanoic acid (28%)(B:-)(C:+)	2,3−Dihydroxybutanoic acid (17%)(B:-)(C:+)
LC-MS (AbsQuant)	Cystine (89%)(B:-)(C:-)<br>3HBA (68%)(B:+)(C:+)<br>Glutamic acid (49%)(B:+)(C:+)<br>Choline (21%)(B:+)(C:+)	Cystine (99%)(B:-)(C:-)<br>3HBA (83%)(B:+)(C:+)<br>Glutamic acid (78%)(B:+)(C:+)<br>Pentothenate (28%)(B:-)(C:+)<br>Urate (17%)(B:+)(C:+)<br>Aspartic Acid (12%)(B:+)(C:+)
LC-MS (RelQuant)	Malate (30%) (B:-)(C:+)	
Lipidyzer (composition)	TAG 50:5(FA16:1) (15%)(B:-)(C:-)<br>TAG 44:1(FA12:0) (14%)(B:-)(C:-)	DAG 14:1/18:1 (19%)(B:+)(C:+)
Lipidyzer (concentration)		

Table S22: Metabolites that are selected using **GMUS** among ⩾10% of replications associated with both BC and CRC risks and the direction of their marginal association to these two cancer risks.

Platform	Half Min Imputation	Multiple Imputation
NMR		
GC-MS		
LC-MS (AbsQuant)	Choline (56%)(B:+)(C:+)<br>Glutamic acid (23%)(B:+)(C:+)<br>Cystine (12%)(B:-)(C:-)	Choline (63%)(B:+)(C:+)<br>Glutamic acid (10%)(B:+)(C:+)
LC-MS (RelQuant)		
Lipidyzer (composition)		
Lipidyzer (concentration)		

Table S23: Metabolites that are robustly (⩾50% times selected) associated with BC risks and the direction of their marginal association to the BC risks using BC specific matched controls.

Method	Platform	Half Min Imputation	Multiple Imputation
Lasso	NMR	N−methylnicotinic acid (53%)(+)	
Lasso	Lipidyzer (composition)	TAG 47:0(FA15:0) (54%)(-)	DAG 14:1/18:1 (81%)(+)
Lasso	Lipidyzer (concentration)	DAG 14:1/18:1 (88%)(+)<br>TAG 48:0(FA16:0) (59%)(+)	
Lasso Order	GC-MS	Alpha−ketoglutarate (100%)(-)	
Lasso Order	NMR	N−methylnicotinic acid (53%)(+)	N−methylnicotinic acid (65%)(+)
Lasso Order	LC-MS (AbsQuant)	Cystine (58%)(-)	
Lasso Order	Lipidyzer (composition)	TAG 47:0(FA15:0) (62%)(-)	DAG 14:1/18:1 (99%)(+)
GMUS	NMR	Uracil (95%)(-)<br>Formate (83%)(+)	Uracil (97%)(-)<br>Formate (82%)(+)
GMUS	Lipidyzer (composition)	TAG 52:2(FA18:2) (60%)(+)	

Table S24: Metabolites that are robustly (⩾50% times selected) associated with CRC risks and the direction of their marginal association to the CRC risks using CRC specific matched controls.

Method	Platform	Half Min Imputation	Multiple Imputation
Lasso	LC-MS (AbsQuant)	3HBA (66%)(+)<br>Cystine (60%)(-)	
Lasso	LC-MS (RelQuant)	Adenosine (52%)(-)	Adenosine (90%)(-)
Lasso	Lipidyzer (composition)		TAG 48:5(FA18:3) (80%)(+)<br>DAG 14:1/18:1 (78%)(+)
Lasso Order	NMR	N−methylnicotinic acid (86%)(-)	N−methylnicotinic acid (94%)(-)
Lasso Order	LC-MS (AbsQuant)	3HBA (95%)(+)<br>Cystine (89%)(-)	3HBA (98%)(+)<br>Cystine (89%)(-)
Lasso Order	LC-MS (RelQuant)		Adenosine (90%)(-)
Lasso Order	Lipidyzer (composition)		TAG 48:5(FA18:3) (80%)(+)<br>DAG 14:1/18:1 (76%)(+)
GMUS	LC-MS (AbsQuant)	Choline (67%)(+)	
GMUS	LC-MS (RelQuant)		Adenosine (90%)(-)
GMUS	Lipidyzer (composition)		PC 16:0/18:2 (55%)(-)

Table S25: Metabolites that are robustly (⩾50% times selected) associated with both BC and CRC risks and the direction of their marginal association to these two cancer risks using specific cancer controls.

Method	Platform	Half Min Imputation	Multiple Imputation
Lasso	Lipidyzer (composition)		DAG 14:1/18:1 (58%)(B:+)(C:+)
Lasso Order	NMR		N−methylnicotinic acid (56%)(B:+)(C:-)
Lasso Order	LC-MS (AbsQuant)	Cystine (89%)(B:-)(C:-)<br>3HBA (68%)(B:+)(C:+)	Cystine (52%)(B:-)(C:-)
GMUS	LC-MS (AbsQuant)	Choline (56%)(B:+)(C:+)	
